# Supplementary material for: Shifting landscapes of human MTHFR missense-variant effects
Source: Am J Hum Genet. 2021 Jul 1;108(7):1283–300. doi: 10.1016/j.ajhg.2021.05.009 (PMC8322931; doi:10.1016/j.ajhg.2021.05.009)
Supplement: Document S1. Figures S1–S3, Figures S5–S7, Table S1, and supplemental methods [file mmc1.pdf]

The American Journal of Human Genetics, Volume *108*

Supplemental information

Shifting landscapes of human MTHFR missense-variant effects

Jochen Weile, Nishka Kishore, Song Sun, Ranim Maaieh, Marta Verby, Roujia Li, Iosifina Fotiadou, Julia Kitaygorodsky, Yingzhou Wu, Alexander Holenstein, Céline Bürer, Linnea Blomgren, Shan Yang, Robert Nussbaum, Rima Rozen, David Watkins, Marinella Gebbia, Viktor Kozich, Michael Garton, D. Sean Froese, and Frederick P. Roth

Supplemental figures

A

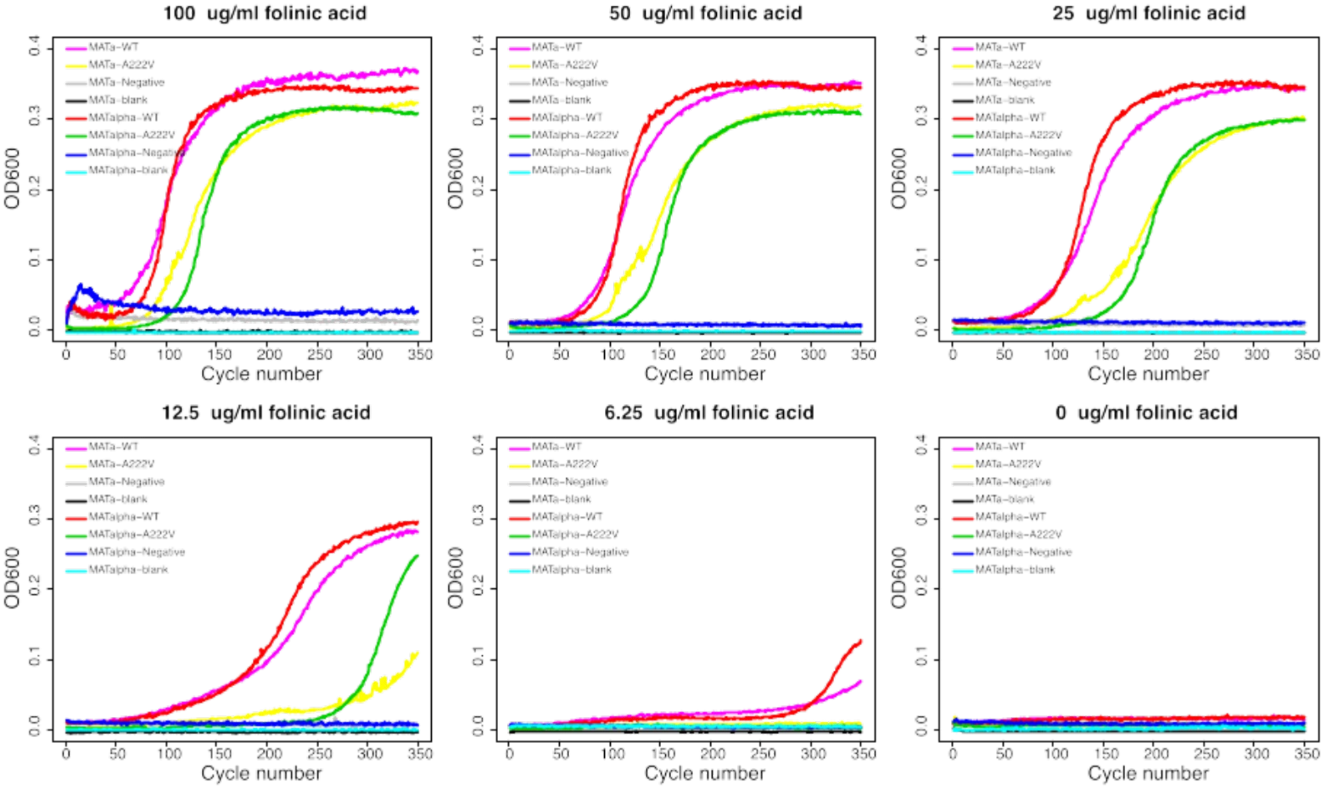

B

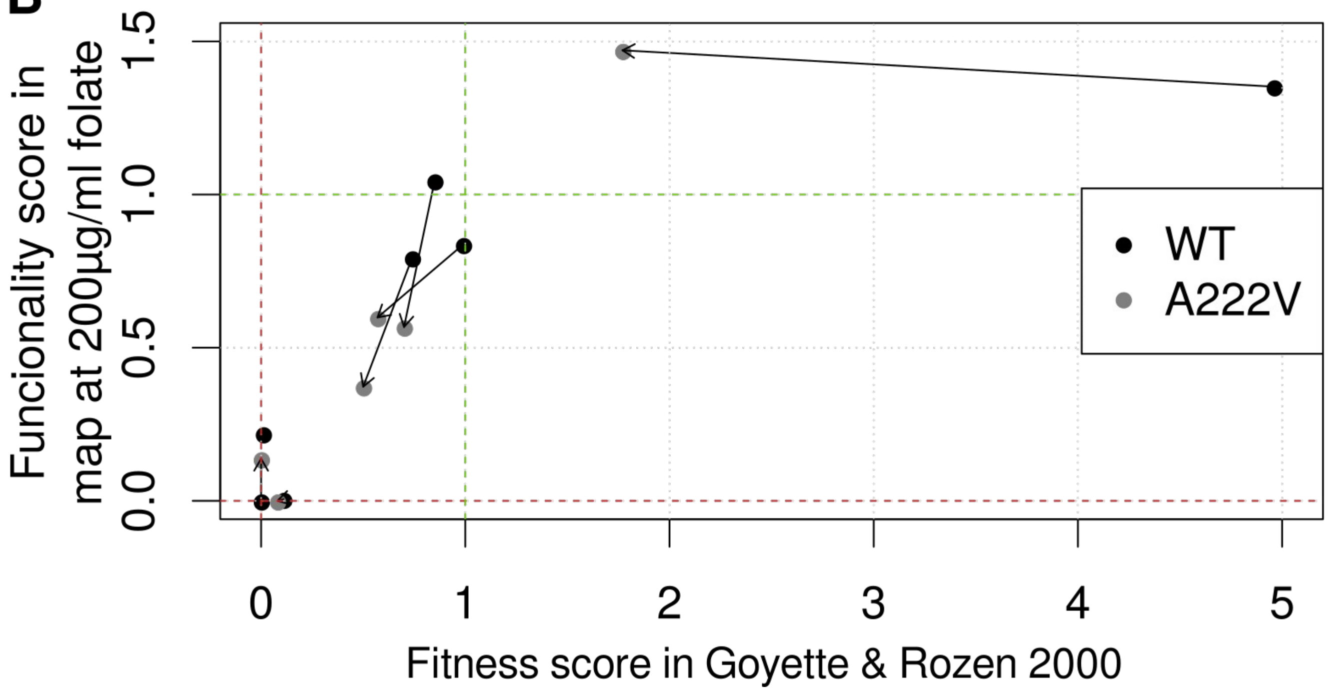

Supplemental Figure S1: A: Assay validation via liquid growth assay. Continuous optical density readings at λ=600nm for liquid growth assays of yeast met13-fol3 strains carrying WT human MTHFR,

the p.Ala222Val variant, as well as an empty vector and empty media control at different levels of folinic acid supplementation. p.Ala222Val was confirmed to exhibit a mild functionality defect compared to WT, which increases in severity as the folinic acid concentration decreases. B: Comparison of VE map scores against previous results for relative enzyme activity in WT and p.Ala222Val backgrounds by Goyette and Rozen 2000. Arrows connect corresponding variants in the two backgrounds. Red and green lines correspond to null-like and WT-like functionality, respectively.

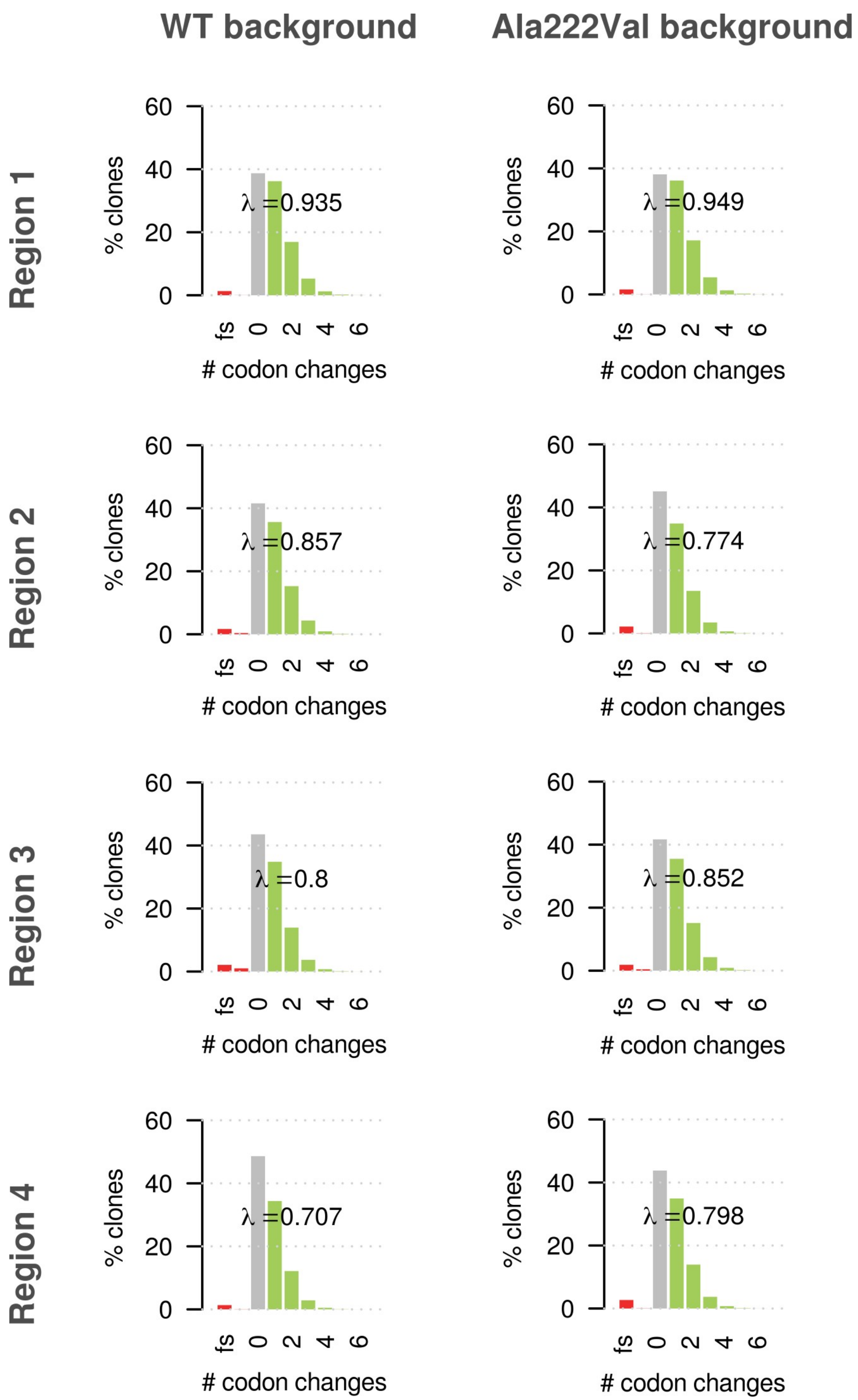

Supplemental Figure S2: Distribution of the number of codon changes across clones in each of the mutagenized libraries. The two red bars on the left indicate the percentage of clones carrying potential frame-shifting and in-frame indels respectively. The Gray bar indicates the percentage of WT clones, while the green bars show clones carrying one or more codon changes. Lambda indicates the

parameter of the best-fitting Poisson distribution over the distribution of codon changes and can be interpreted as the average number of amino acid changes per clone.

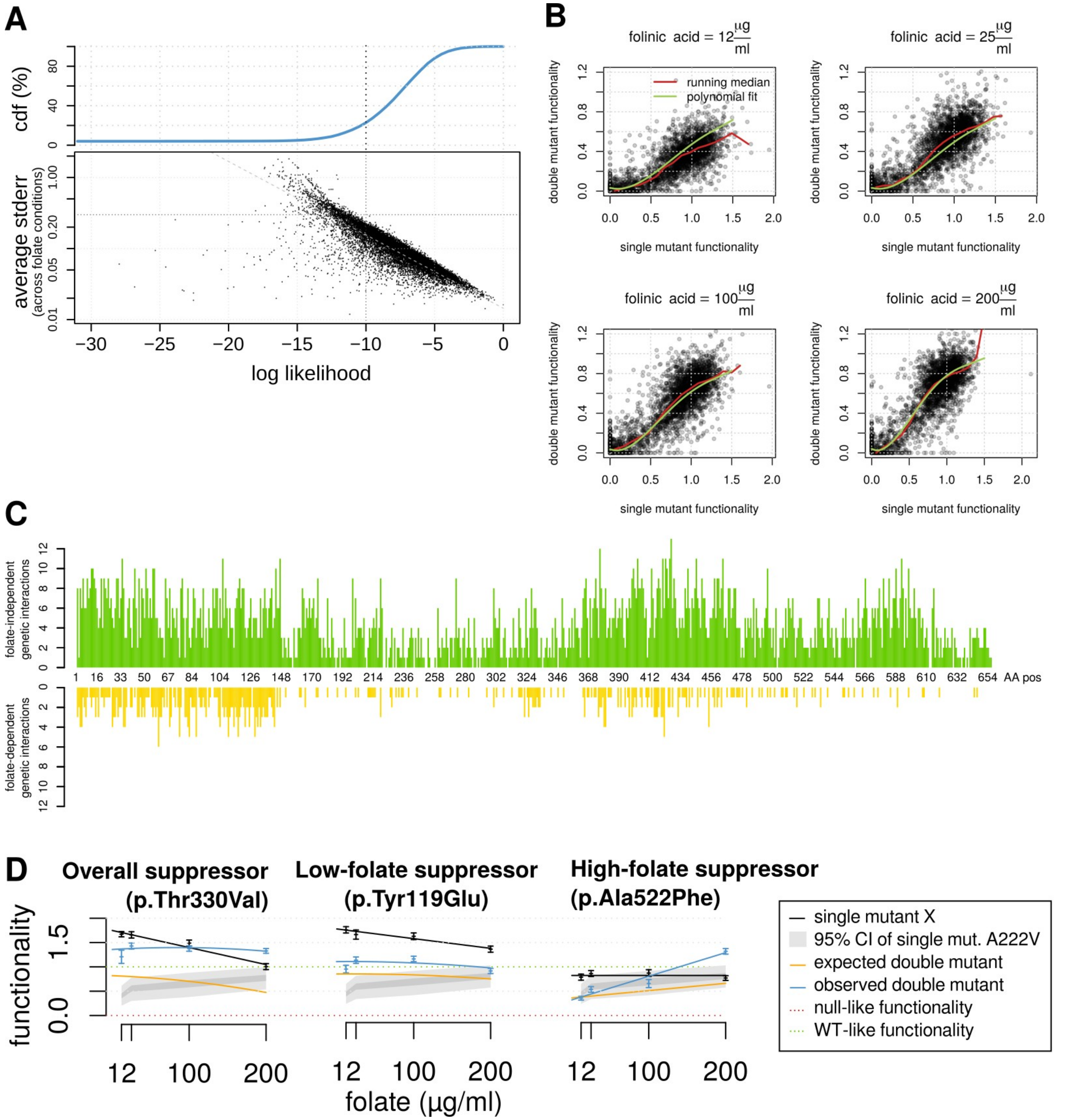

Supplemental Figure S3: A: Cumulative distribution of model log likelihoods and scatterplot comparing log likelihood against the average SEM of modeled data points. B: Running medians and polynomial regression fits for single- vs double-mutant functionality scores at each tested folinate concentration. These form the basis for the “expected double mutant functionality” used to find genetic interactions. C: Number of folate-independent (green) and folate-dependent (yellow) genetic interactions per amino acid position. D: Example variants for three different categories of suppressors of p.Ala222Val. Left: Folate-independent suppressors; Middle: Suppressors at low folate levels; Right:

Suppressors at high folate levels. The black line in each plot shows the single mutant functionality model of the variant in question, with the underlying data points and their SEM indicated by black bars, the gray areas indicate the 95% confidence interval of the p.Ala222Val single mutant functionality based on the distribution of synonymous variants in that background. The orange line indicates the expected double mutant functionality under the regression model. The blue line indicates the best fitting model for the double mutant functionality with the underlying data points and their respective SEM shown as blue bars.

Supplemental Figure S4: Full-sized maps, colors and labels as in Figure 2. See external file MTHFR_map_suppl_fig_S4.png

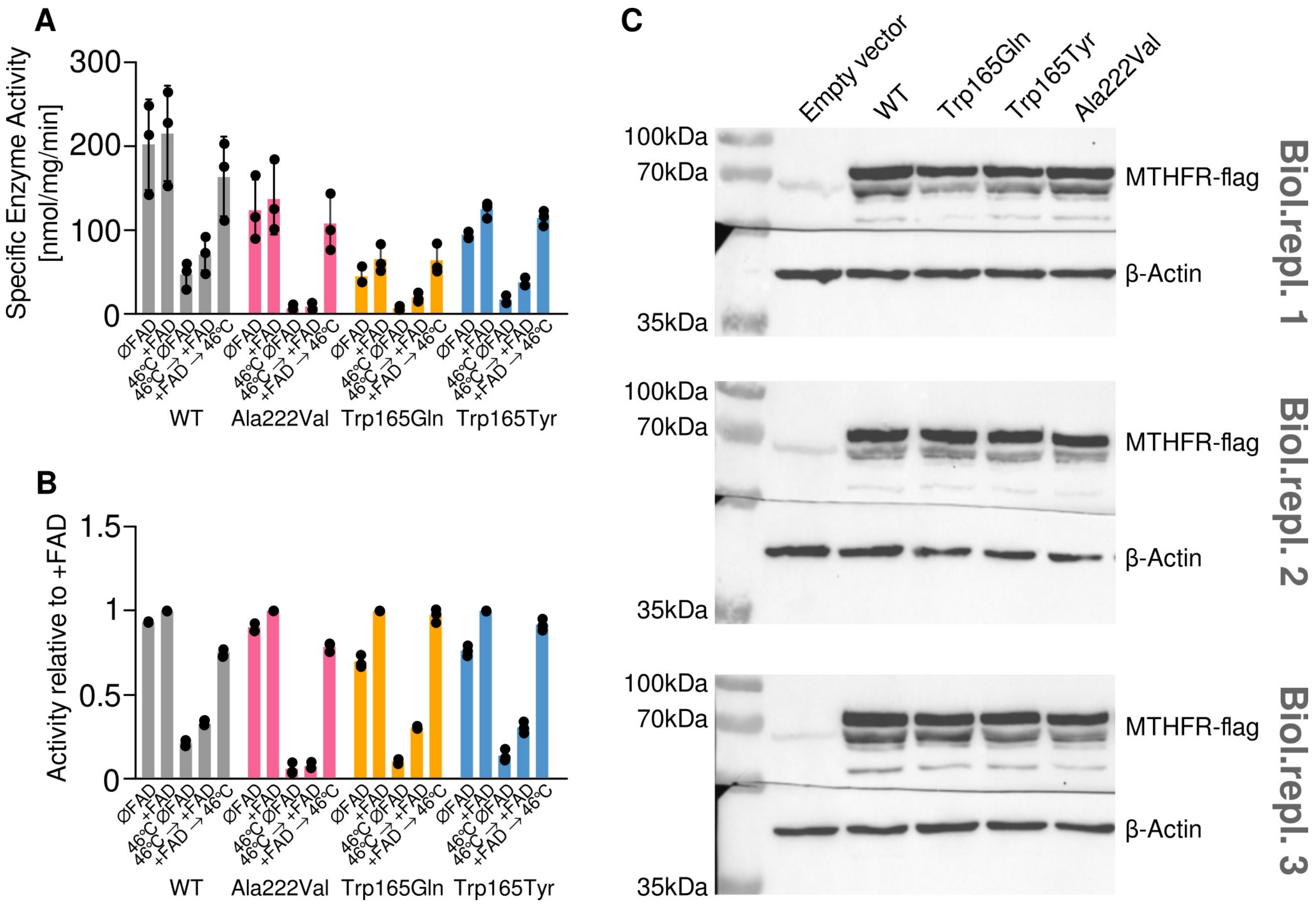

Supplemental Figure S5: Specific enzyme activity as measured by HPLC with cell lysates derived from MTHFR-KO cells following transfection with variant MTHFR vectors. Activity was assayed in the presence and absence of FAD, as well as in three heat treatment conditions: Without FAD, with FAD supplementation before heat treatment and FAD supplementation after heat treatment. (A) Absolute specific activity. Error bars show standard deviation. (B) Activity normalized relative to +FAD condition. Error bars show standard deviation. (C) Western blots confirm the expression levels of the tested variants.

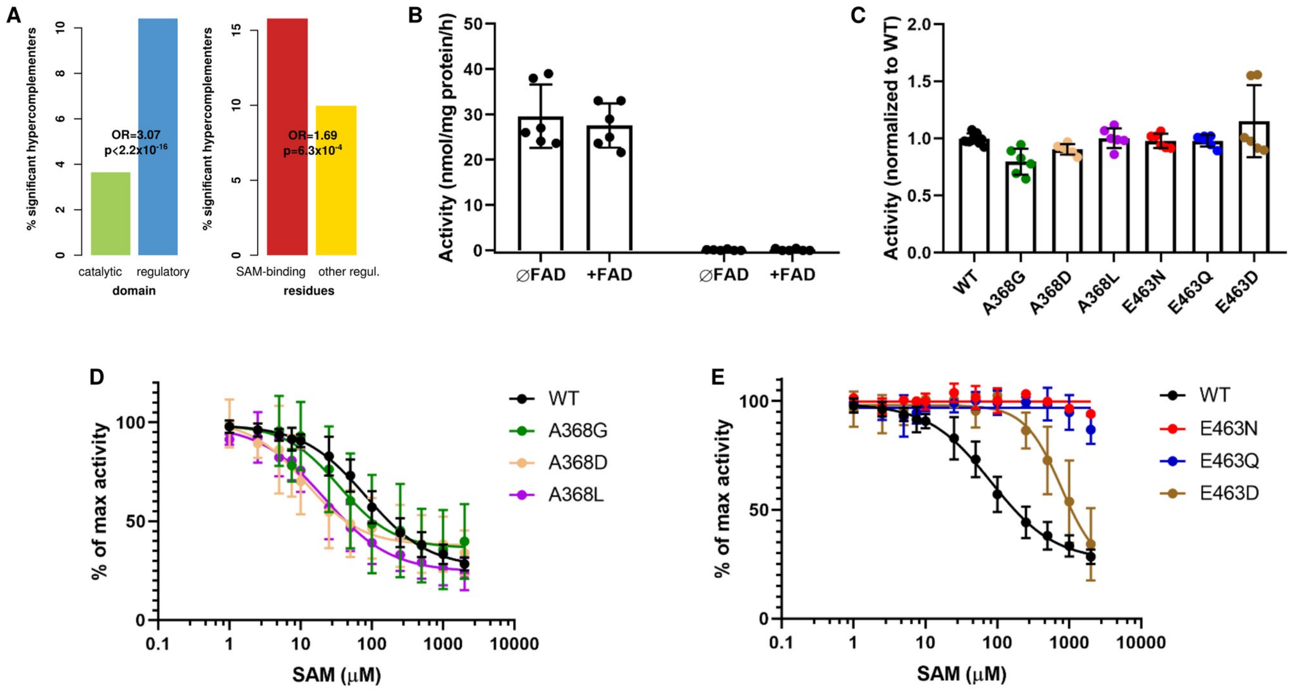

Supplemental Figure S6: A. Fisher's exact tests for enrichment of hypercomplementers in the regulatory domain, as well as enrichment of hypercomplementers in residues near the SAM binding interface. B. Activity of endogenous MTHFR from cell lysates of unmodified HEK293T (ATCC: CRL-3216, WT) cells and genetically engineered MTHFR knock-out HEK293 cells (MTHFR-KO). Assay was performed in the absence (ØFAD) and presence (+FAD) of 75 µM FAD supplemented to the reaction mixture. n = 3 biological replicates, performed in duplicate. C. Maximum activity of MTHFR from cell lysates derived from MTHFR-KO cells following transfection with WT or mutant MTHFR vectors. To account for activity variability following transfection (REF: Burda et al. J Inherit Metab Dis 2016), activity is normalized to WT for each of n = 3 biological replicates (performed in duplicate). D and E. SAM inhibition of MTHFR from cell lysates derived MTHFR-KO cells following transfection with WT or mutant vectors. n = 3 biological replicates (performed in duplicate). Ki's: WT: 82.4 ± 10.4 µM; A368G: 35.0 ± 16.4 µM; A368D: 37.8 ± 4.0 µM; A368L: 24.3 ± 5.3 µM; E463N: n.d.; E463Q: n.d.; E463D: n.d. where n.d. means not determinable. Error bars in B-E show standard deviation.

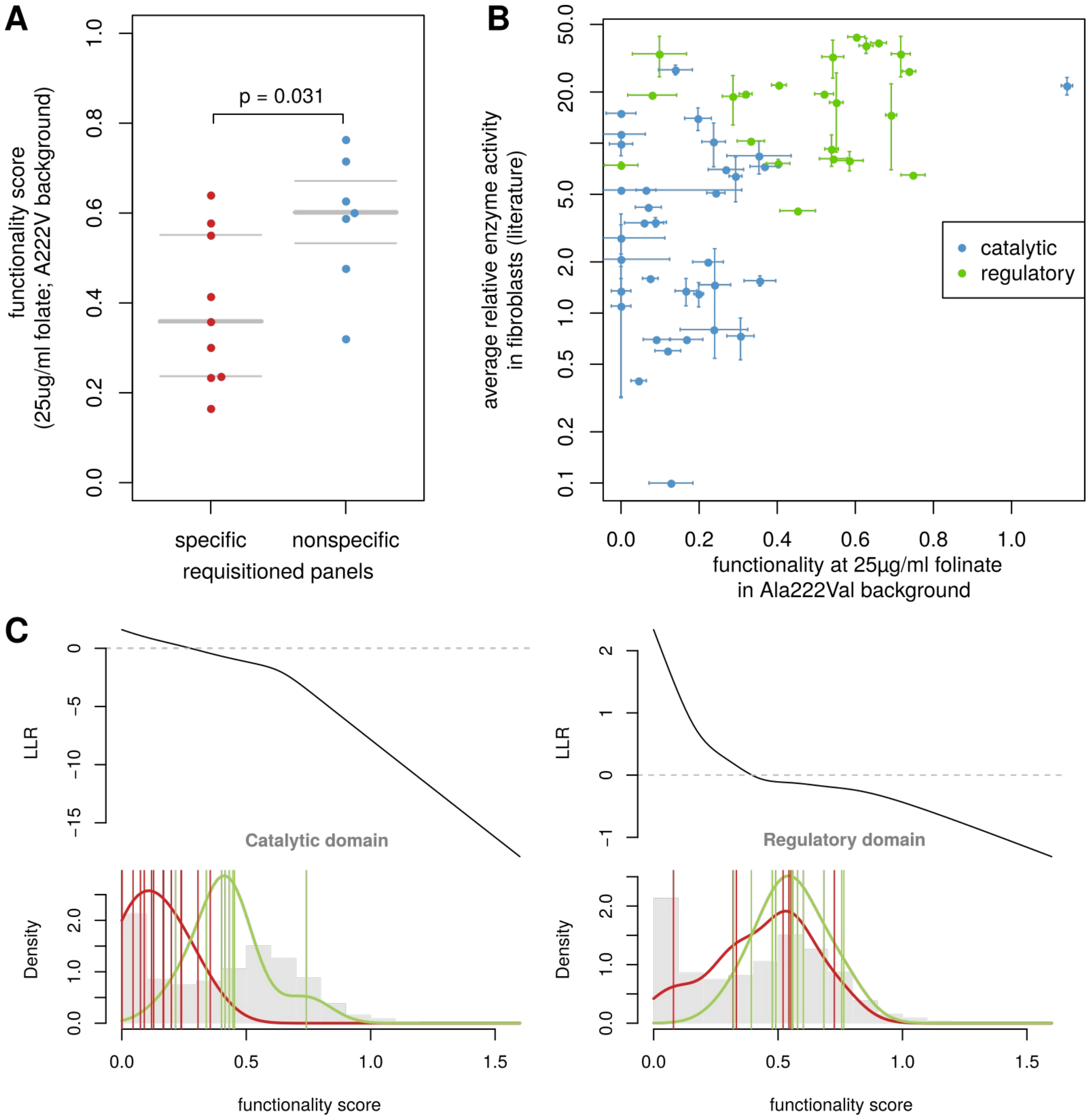

Supplemental Figure 7: A: Functionality scores in the VE map for 25µg/ml folate in the p.Ala222Val background for variants observed by Invitae in disease-specific and non-specific panels. p-value shown for Mann-Whitney U-test. B: Functionality at 25µg/ml folinic acid in the p.Ala222Val background correlates with average enzymatic activity in fibroblasts as reported in the literature (see Methods). Error bars show standard deviation. Blue data points are catalytic domain variants, while green data points are regulatory domain variants. C: Transformation functions from functionality scores to log likelihood ratios of pathogenicity in the catalytic and regulatory domains. The functions express the log ratio between the likelihood of observing a given score in the score distribution of the positive reference (red) set as opposed to that of the negative reference set (green). Gray histogram bars show the distribution of missense variants for comparison.

Supplemental Tables

**Background**	**folinic acid (µg/ml)**	**Possible AA variants**	**detected**	**%**	**passed filter**	**%**
WT	12.5	13776	13190	95.75%	12534	90.98%
WT	25	13776	13189	95.74%	12494	90.69%
WT	100	13776	13191	95.75%	12530	90.96%
WT	200	13776	13142	95.40%	12551	91.11%
p.Ala222Val	12.5	13776	12970	94.15%	12011	87.19%
p.Ala222Val	25	13776	12970	94.15%	11997	87.09%
p.Ala222Val	100	13776	12964	94.11%	12061	87.55%
p.Ala222Val	200	13776	12916	93.76%	12158	88.25%

Supplemental Table S1: Filter pass rates for all possible AA change, synonymous and nonsense variant across all 8 conditional maps. Detected: Variants for which at least one sequencing read was detected. Passed filter: Variants that passed the quality filtering procedure (see Methods for details) and were accepted into the map.

Supplemental Table S2: Variant effect data and models of functionality, folinate response and genetic interactions. See file MTHFR_map_suppl_tableST2.xlsx

Supplemental Table S3: Curated reference variant sets for validation. See file MTHFR_map_suppl_tableST3.xlsx

Supplemental Table S4: List of TileSeq primers and PopCode mutagenesis oligos. See file MTHFR_map_suppl_tableST4.xlsx

Supplemental Methods

A. Analysis of sequence data to derive raw functionality scores

After consolidating variants by amino acid change outcome, variants with a frequency of pre-selection reads that fell below three standard deviations above the replicate mean of the corresponding non-mutagenized control were considered to have a frequency indistinguishable from that arising due to PCR or base-calling errors and filtered out. Similarly, variants with fewer post-selection read counts than this threshold were considered lost in a culture propagation bottleneck and also filtered out. The dataset was split into sets of variants falling into the original mutagenesis regions, so they could be rescaled separately from each other. Frequencies in the non-mutagenized control frequencies were then subtracted from pre- and post-selection frequencies to adjust for sequencing cycle-specific error biases. The raw functionality scores were then calculated as the log ratio between the corrected pre- and post selection counts. The final scores were then calculated by adjusting the functionality scores to a 0 to 1 scale, where 0 corresponds to the median score of nonsense variants (assumed to be complete loss of function) and 1 corresponds to the median score of synonymous variants (assumed to be of WT-like function). Importantly, this is done for each mutagenesis region separately, to ensure that they fall on the same scale.

Measurement uncertainty was determined by using a method by Baldi and Long[55] to regularize the empirical coefficient of variation across technical replicates using a prior obtained from linear regression against the raw read counts. Error was propagated through each subsequent transformation operation using Taylor approximations. The full code for performing these steps can be found on Github at https://github.com/jweile/tileseqMave (commit number fa8b190).

B. Modeling dependence of variant effects on folinate and p.Ala222Val

Given only four data points per variant (i.e. the measurements taken at the four chosen folinate concentrations), modeling functionality in terms of folinate supplementation required a parsimonious approach. Evaluating both linear and sigmoid models using Akaike's Information Criterion (AIC), we found 96.1% of variants to have a more favorable (lower) AIC under the linear model. Manual inspection of maximum-likelihood sigmoid fits also revealed that these tended to either mimic linear behavior or assume extreme step-like shapes.

Given these results, the response of each variant (i) to folinate supplementation was modeled using a simple linear function that expresses functionality at concentration c in terms of a base functionality (b) and a folinate response parameter (r).

$$f_i^{(sm)}(c) = b_i + r_i c$$

The likelihood of a given model can be determined as the product of densities under normal distributions based on the sample means and standard deviations of the experimental measurements ($\hat{f}_{i,c}$ and $\hat{\sigma}_{i,c}$) at the four given folinate concentrations:

$$\mathcal{L}\left(f_i^{(sm)}(c)\right) = \prod_{c\in\{12.5,25,100,200\}} \varphi_{\mu=\hat{f}_{i,c},\sigma=\hat{\sigma}_{i,c}}\left(f_i^{(sm)}(c)\right)$$

We determined the maximum likelihood model for each model using the Nelder-Mead algorithm implemented in the "optimization" R package[1,2].

To find a threshold for acceptable model quality, we compared the log likelihood against the average experimental standard error across conditions. We found that a model log likelihood of -10 roughly corresponded to a maximal standard error of 0.2 in terms of functionality (where a unit of 1 represents the difference between WT and complete loss of function).

To assess the plausibility of each response model we compared it with the corresponding null-model, which assumes no supplementation response (calculating the likelihood that the true functionality is constant at the mean of all four measurements, given their respective measurement error). This null-model likelihood allows for the determination of a log likelihood ratio (LLR_s) expressing how much more likely the supplementation response model is compared to the null model. To correct for multiple hypothesis testing and limit the false positive rate, we applied a prior probability of 1% and selected those cases in which the LLR_s transformed this prior to a posterior probability of greater than 99%.

To model dependence of each variant on the p.Ala222Val background, a similar model was used. We modeled the expectation for double mutant functionality $f^{(edm)}$ as a third order polynomial spline interpolation between single and double-mutant fitness given each folinate concentration and the functionality of p.Ala222Val itself at the same concentration. Folinate-independent and folinate-dependent genetic interactions were then modeled as additive parameters $\varepsilon^{(b)}$ and $\varepsilon^{(r)}$:

$$f_i^{(dm)}(c) = f^{(edm)}\left(f_i^{(sm)}, f_{\mathrm{A222V}}^{(sm)}, c\right) + \varepsilon_i^{(b)} + \varepsilon_i^{(r)}c$$

Model likelihood was calculated as for the single mutant functionality scores. Plausibility of models was evaluated against two different null models, one without genetic interactions (i.e. $\varepsilon^{(b)} = \varepsilon^{(r)} = 0$), and the other modeling only folinate-independent genetic interactions (i.e. $\varepsilon^{(r)} = 0$). LLR_g values were calculated as above for LLR_s.

C. Molecular dynamics simulation of the FAD binding site

Using above-described structural model (created from PDB:6FCX and PDB:2FMN) we used Amber Modeller 9.24 to add the amino acids for the missing disordered loop across positions 159-174. We created six different models, with residue 165 represented as either the WT tryptophan, or a mutant aromatic tyrosine or a mutant polar glutamine; each in the presence and absence of a docked LY309887 (a folate analogue) at the active site. The Antechamber

package[3] was used to generate topology and coordinate files containing FAD and LY309887. Using Amber18[4], we neutralized the models using Cl^- and Na^+ ions and solvated using the TIP3P water model (buffer distance 12Å). Using periodic boundary conditions, we then sequentially performed steepest-descent and conjugate-gradient-energy minimization, followed by equilibration molecular dynamics simulations, gradually removing constraints and heating from 0 to 300K. Unconstrained molecular dynamics simulations were then performed on the equilibrated systems using Amber18. We calculated 10 replicate trajectories for each model, across a timeframe of 200 ns with a resolution of 0.2 ns.

We then calculated the distance between the alpha carbon atom of amino acid 165 (i.e. the WT Trp, or mutant Tyr or Gln) and the C1 carbon of the FAD flavin (using Euclidean distance at each simulation time point). We next calculated the distance of between FAD's central N5 atom from the center of the top of the catalytic domain's TIM-barrel (defined as as the arithmetic centroid between the alpha carbons of residues 321, 256, 227, 196, 156, 129, 93, and 64), also at every time point. These distance trajectories were then used to calculate the time spent at given distances.

To generate the Markov Model of interaction states between Trp165 and the FAD flavin, we calculated the relative transposition vector and relative rotation quaternion between the two molecules' respective aromatic rings in each time point and then used Mclust[5] to perform Gaussian mixture model clustering, identifying 8 distinct clusters. We calculated the centroid of each cluster and visualized the timepoints with the greatest similarity to each centroid using OpenPyMol[6]. Finally, we examined the pattern in which the simulation trajectories traversed through cluster members and calculated lingering times and transition probabilities. These were then used to construct the state transition model.

D. Compilation of reference variant sets

To evaluate the ability of map scores to predict variant pathogenicity (and ultimately individual phenotypes), we needed to establish positive and negative variant reference sets. Hyperhomocysteinemia case genotypes and phenotypes were assembled largely from previous publications[7,8]. For all individuals for which data had not been published previously: clinical, biochemical, and molecular genetic data were obtained during routine care; individuals gave their informed consent for DNA analysis; and phenotypes were collected within a research project after obtaining informed consent, which included also a consent for the publication of clinical, enzymatic, and molecular genetic data. For individuals followed in the Metabolic Center in the Department of Pediatrics and Adolescent Medicine, the General University Hospital in Prague, there was approval of the Ethics Committee (1194/13 S-IV). All data collection conformed to the principles of the Helsinki Declaration.

We integrated these datasets to match the most recent MTHFR reference sequence and converted the associated phenotype data to use uniform time units for age of onset. The dataset (Supplemental Table S3A) comprises 206 samples, 197 and 128 of which are labeled approximate and detailed age of onset, respectively. All but two samples provide genotype

information, with 89 of them carrying missense variants in both alleles. However only 78 samples provide information on p.Ala222Val status.

We extracted the list of missense variants in the above dataset and tallied how often they each occured in early- and late-onset cases respectively. Following existing convention, the classification cutoff for early onset was defined as diagnosis no later than 12 months of age[7,8]. Variants that were seen more often in early onset than late onset cases were included in the "early onset positive reference set" (comprising 30 variants); while variants that were more often observed in late onset cases than early onset cases were included in the "late onset reference set" (comprising 40 variants). Ties were excluded.

Next, to obtain a random reference cohort, we accessed gnomAD[9,10] (a collection of genotypes meant to be comprised primarily of unaffected individuals), filtering for missense variants in MTHFR that fulfilled either one of the two following criteria to enrich for variants likely to be benign: (i) The global minor allele frequency is above 1 in 10,000; or (ii) at least one homozygous case has been observed. Within this set of variants set we found that two gnomAD entries (c.1408G>C = p.Glu470Gln and c.1409A>T = p.Glu470Val) were actually a mis-annotated multi-nucleotide variant (c.1408_1409delinsCT = p.Glu470Leu). We therefore replaced these variants with the correct MNV in our resulting random reference set. We also removed p.Ala222Val, as it was already used here as a common genetic background for our maps.

E. Sequencing panel enrichment analysis

We examined MTHFR variants previously observed in clinical sequencing by Invitae. Although phenotypes are not available for sequenced individuals, it is known which gene panel was requested by the physician for sequencing. Variants were stripped of all protected health information (i.e. de-identified) under an approved protocol from the Western Institutional Review Board (IRB #20161796). Panel-associations were collated for each unique variant and were classified into two categories: Relevant disease-specific panels (homocystinuria, fatty-acid oxidation defects and neurometabolic disease) and Nonspecific panels (such as carrier screening). We then grouped variants according to whether they were more often seen in specific or nonspecific panels and compared the distributions of corresponding functionality values from our atlas (using the 25µg/ml folinic acid and p.Ala222Val background map) via Mann-Whitney-U test.

F. Determining a log likelihood ratio of pathogenicity for each variant

The evaluation against reference sets showed that functionality scores in the catalytic and regulatory domains were not on a comparable scale in terms of predicting disease. After all, the numerical values in our atlas relate to to the fitness of yeast cells as a result of the degree of functionality of the human variant in the host pathway. Therefore, we implemented a transformation function to represent variant effects in terms of the strength of evidence towards and against pathogenicity, that is, a log likelihood ratio of pathogenicity.

To this end we used the distributions of functionality scores for the positive and random reference sets in both domains in the best-performing map (p.Ala222Val background at 25µg/ml folinate) and the best-performing metaparameter for the linear model (WT background at 120µg/ml folinate) to construct likelihood ratios. For any given score, we calculate how much more (or less) likely it is to observe it under the distribution of the positive reference set than under the distribution of the random reference set. We determined the probability density functions for functionality scores in the positive (early onset) reference variants and negative (gnomAD) reference variants in each domain using kernel density estimation via the R package kdensity[11]. The log likelihood ratio (LLR_p) transformation for a given functionality f was then defined as:

$$LLR_p(f) = \log\left(\frac{\pi_X(f)}{\pi_Y(f)}\right)$$

, where π_X is the probability density of the positive reference set distribution and π_Y is the probability density of the random reference set distribution.

G. Modeling individualized diploid genotypes

The first model (M_1) interprets variants as if they were in the WT background; the second model, (M_2) accounts for p.Ala222Val. The third model (M_3) accounts for an additional common variant, p.Glu429Ala (E429A). All models transform functionality scores to pathogenicity LLRs and then use the minimum LLR across variants occurring in trans (in keeping with the recessive mode of inheritance)

$$M_1 = \min_{i\in\{\text{left},\text{right}\}} (LLR_p(f_i^{(sm)}))$$

$$M_2 = \min_{i\in\{\text{left},\text{right}\}} \left(LLR_p\left(\begin{cases}\text{WT}: & f_i^{(sm)}\\ \text{A222V}: & f_i^{(dm)}\end{cases}\right)\right)$$

$$M_3 = \min_{i\in\{\text{left},\text{right}\}} \left(LLR_p\left(\begin{cases}\text{WT}: & f_i^{(sm)}\\ \text{A222V}: & f_i^{(dm)}\\ \text{E429A}: & f_i^{(sm)} \times f_{\text{E429A}}^{(sm)}\\ \text{A222V\&E429A}: & f_i^{(dm)} \times f_{\text{E429A}}^{(dm)} \times \frac{1}{f_{\text{A222V}}^{(sm)}}\end{cases}\right)\right)$$

H. Prior-balancing and significance testing for precision-recall analysis

The precision observed at a given score threshold is defined as the number of true positive calls divided by the total number of positive calls. As the threshold increases in stringency, the

total number of positives decreases, leading to reduced numerical stability and increased uncertainty for precision estimates. This can be expressed as a Bernoulli process, governed by a binomial distribution for*i* true positives out of *n* total positives:

$$\mathbb{P}(i|\rho) = \binom{n}{i}\rho^i(1-\rho)^{(n-i)}$$

Thus, if we model a true precision ρ that is lower than x, then the likelihood of the observation is:

$$\mathbb{P}(i|\rho < x) = \int_0^x \binom{n}{i}\rho^i(1-\rho)^{(n-i)}d\rho$$

However, since we are interested in the CDF of the posterior P(ρ<x|i), we need to use Bayes' theorem.

$$\mathbb{P}(\rho < x|i) = \frac{\mathbb{P}(i|\rho < x)\mathbb{P}(\rho < x)}{\mathbb{P}(i)}$$

Then, using a uniform prior:

$$\mathbb{P}(\rho < x|i) = \frac{\int_0^x \binom{n}{i}\rho^i(1-\rho)^{(n-i)}d\rho \cdot x}{\int_0^1 \binom{n}{i}\rho^i(1-\rho)^{(n-i)}xd\rho} = \frac{\int_0^x \binom{n}{i}\rho^i(1-\rho)^{(n-i)}d\rho}{\int_0^1 \binom{n}{i}\rho^i(1-\rho)^{(n-i)}d\rho}$$

To estimate p-values for significant differences in AUPRC between two predictors, we can calculate the CDF for the reference predictor as above, then calculate the AUC at each quantile of the above distribution and look up the AUC of the second predictor in the CDF of the first. This yields the probability of observing an AUC at least as extreme. An R-implementation of this can be found at https://github.com/jweile/yogiroc.

To enforce the tendency for increasingly stringent thresholds to yield increased precision (at the cost of recall), we apply a 'monotonization' function to the PRC curve, such that precision levels (and confidence interval traces) observed at a given stringency can only rise or stay constant as stringency is further increased.

Imbalances in the sample sizes of the positive and negative reference sets lead to different underlying prior probabilities (of pathogenicity in this case). Varying priors make it difficult to compare precision recall curves with one another, as precision of a prediction is a function of both the strength of evidence and the prior probability that the prediction will be correct. We therefore generated balanced precision vs. recall curves as in Wu et al (under review), via a procedure derived from Bayes' Rule. Briefly, if *ρ* is the precision at a given score threshold, and *P* the prior probability, then the balanced precision is:

$$\rho_{\text{balanced}} = \frac{\rho(1-P)}{\rho(1-P)+(1-\rho)P}$$

Supplemental references

1. Nelder, J.A., and Mead, R. (1965). A Simplex Method for Function Minimization. Comput. J. *7*, 308–313.

2. Husmann, K., Lange, A., and Spiegel, E. (2017). The R Package optimization : Flexible Global Optimization with Simulated-Annealing. p.

3. Wang, J., Wang, W., Kollman, P.A., and Case, D.A. (2006). Automatic atom type and bond type perception in molecular mechanical calculations. J. Mol. Graph. Model. *25*, 247–260.

4. Case, D.A., Cheatham, T.E., Darden, T., Gohlke, H., Luo, R., Merz, K.M., Onufriev, A., Simmerling, C., Wang, B., and Woods, R.J. (2005). The Amber biomolecular simulation programs. J. Comput. Chem. *26*, 1668–1688.

5. Scrucca, L., Fop, M., Murphy, T.B., and Raftery, A.E. (2016). mclust 5: Clustering, Classification and Density Estimation Using Gaussian Finite Mixture Models. R J. *8*, 289–317.

6. Schrödinger (2016). The PyMOL Molecular Graphics System.

7. Froese, D.S., Huemer, M., Suormala, T., Burda, P., Coelho, D., Guéant, J.-L., Landolt, M.A., Kožich, V., Fowler, B., and Baumgartner, M.R. (2016). Mutation Update and Review of Severe Methylenetetrahydrofolate Reductase Deficiency. Hum. Mutat. *37*, 427–438.

8. Ueland, P.M., and Rozen, R. (2005). MTHFR Polymorphisms and Disease (CRC Press).

9. Lek, M., Karczewski, K.J., Minikel, E.V., Samocha, K.E., Banks, E., Fennell, T., O'Donnell-Luria, A.H., Ware, J.S., Hill, A.J., Cummings, B.B., et al. (2016). Analysis of protein-coding genetic variation in 60,706 humans. Nature *536*, 285–291.

10. Karczewski, K.J., Francioli, L.C., Tiao, G., Cummings, B.B., Alföldi, J., Wang, Q., Collins, R.L., Laricchia, K.M., Ganna, A., Birnbaum, D.P., et al. (2019). Variation across 141,456 human exomes and genomes reveals the spectrum of loss-of-function intolerance across human protein-coding genes. BioRxiv 531210.

11. Moss, J., and Tveten, M. (2019). kdensity: Kernel Density Estimation with Parametric Starts and Asymmetric Kernels.
