# Supplementary figures and images for: Shifting landscapes of human MTHFR missense-variant effects
Source: Am J Hum Genet. 2021 Jul 1;108(7):1283–300. doi: 10.1016/j.ajhg.2021.05.009 (PMC8322931; doi:10.1016/j.ajhg.2021.05.009)

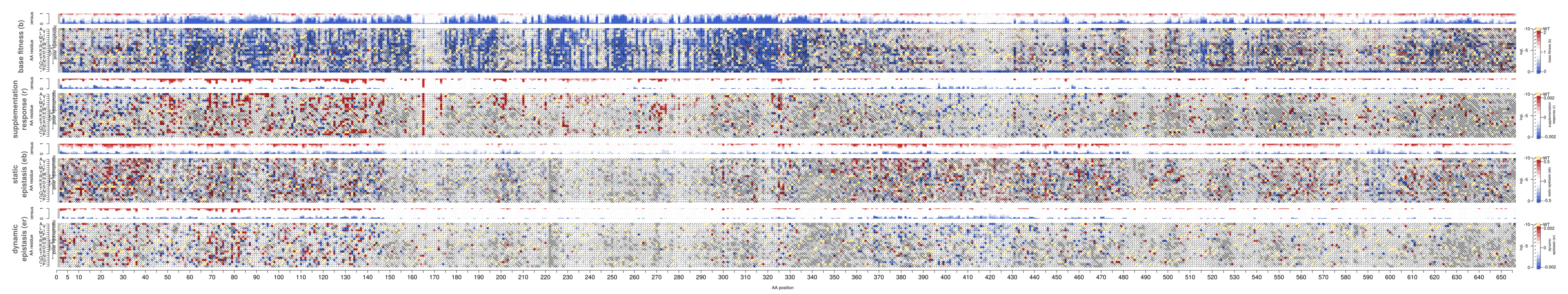

Supplement: Figure S4. Full-sized maps — Colors and labels are as in Figure 2. [file mmc2.pdf]
