# Supplementary material for: Shifting landscapes of human MTHFR missense-variant effects
Source: Am J Hum Genet. 2021 Jul 1;108(7):1283–300. doi: 10.1016/j.ajhg.2021.05.009 (PMC8322931; doi:10.1016/j.ajhg.2021.05.009)
Supplement: Document S2. Article plus supplemental information [file mmc6.pdf]

Shifting landscapes of human MTHFR missense-variant effects

Jochen Weile,[1,2,3,4] Nishka Kishore,[1,2,3] Song Sun,[1,2,3,4] Ranim Maaieh,[5] Marta Verby,[1,2,3] Roujia Li,[1,2,3,4] Iosifina Fotiadou,[1,2,3] Julia Kitaygorodsky,[1,2,3] Yingzhou Wu,[1,2,3,4] Alexander Holenstein,[6] Céline Bürer,[6] Linnea Blomgren,[6] Shan Yang,[7] Robert Nussbaum,[7] Rima Rozen,[8] David Watkins,[8] Marinella Gebbia,[1,2,3] Viktor Kozich,[9] Michael Garton,[5] D. Sean Froese,[6] and Frederick P. Roth[1,2,3,4,*]

Summary

Most rare clinical missense variants cannot currently be classified as pathogenic or benign. Deficiency in human 5,10-methylenetetrahydrofolate reductase (MTHFR), the most common inherited disorder of folate metabolism, is caused primarily by rare missense variants. Further complicating variant interpretation, variant impacts often depend on environment. An important example of this phenomenon is the MTHFR variant p.Ala222Val (c.665C>T), which is carried by half of all humans and has a phenotypic impact that depends on dietary folate. Here we describe the results of 98,336 variant functional-impact assays, covering nearly all possible MTHFR amino acid substitutions in four folinate environments, each in the presence and absence of p.Ala222Val. The resulting atlas of MTHFR variant effects reveals many complex dependencies on both folinate and p.Ala222Val. MTHFR atlas scores can distinguish pathogenic from benign variants and, among individuals with severe MTHFR deficiency, correlate with age of disease onset. Providing a powerful tool for understanding structure-function relationships, the atlas suggests a role for a disordered loop in retaining cofactor at the active site and identifies variants that enable escape of inhibition by S-adenosylmethionine. Thus, a model based on eight MTHFR variant effect maps illustrates how shifting landscapes of environment- and genetic-background-dependent missense variation can inform our clinical, structural, and functional understanding of MTHFR deficiency.

Introduction

A major challenge in relating genomes to traits is the phenomenon of incomplete penetrance, or "genetic resilience."[1] In humans, it has long been known that the impact of disease-causing variants differs between individuals. For example, 30%–40% of individuals with pathogenic *BRCA1* variants remain free from breast and ovarian cancer throughout their lifetimes.[2] Explanations for incomplete penetrance can include environmental effects that trigger, mediate, or suppress the effects of an allele, e.g., exposure to ionizing radiation[3] or genetic interactions (when one allele has a surprising effect on the impact of another[4,5]). Although the dependence of variant effects on environment and genetic background can be complex, these can often be modeled via cell-based assays.[6]

Clinical variant interpretation,[7,8] which weighs available evidence to classify pathogenicity, places a high weight on the results of functional assays.[7] However, cell-based functional assays are resource intensive and are usually lacking for newly discovered variants, and the majority of all missense variants are classified as variants of uncertain significance (VUS).[9] It is increasingly clear that testing variant functions "reactively" (only after first observing the variant in a human) cannot keep pace. A more "proactive" approach, in which multiplexed assays of variant effect (MAVEs) are applied to systematically test variants in disease-associated genes, is emerging; such variants include missense variants not yet seen in any human,[10,11] e.g., for cystathionine beta-synthase (CBS)[12] and calmodulin.[10]

Here, we examine the human gene *MTHFR*, encoding 5,10-methylenetetrahydrofolate reductase, a key enzyme in the one-carbon metabolism pathway that includes the essential folate and methionine cycles. Severe MTHFR deficiency (MIM: 236250), the most common inherited disorder of folate metabolism, has been described in more than 200 individuals,[13] most of whom were diagnosed in infancy.[14] Primarily a disease of the central nervous system, it can also result in thromboembolism or eye disease.[15] In older individuals, ataxic gait, psychiatric disorders, and symptoms related to cerebrovascular events have been reported.[16,17] Biochemically, severe MTHFR deficiency is associated with massive accumulation of homocysteine in the blood (hyperhomocysteinemia), i.e., plasma homocysteine elevated up to 60–320 μM; reference range typically 5–15 μM) together with low or low-normal plasma methionine.[18] In contrast to severe deficiency, some variants in MTHFR yield mild hyperhomocysteinemia (15–30 μM), which increases the risk of neural-tube birth defects and has been reported to increase the risk of cardiovascular disease,[19,20] although the latter association is controversial,[21,22] and of neural-tube birth defects.[23,24] Dietary

[1]Lunenfeld-Tanenbaum Research Institute, Sinai Health System, Toronto, ON M5G 1X5, Canada; [2]The Donnelly Centre, University of Toronto, Toronto, ON M5S 3E1, Canada; [3]Department of Molecular Genetics, University of Toronto, Toronto, ON M5S 3E1, Canada; [4]Department of Computer Science, University of Toronto, Toronto, ON M5S 2E4, Canada; [5]Institute of Biomedical Engineering, University of Toronto, ON M5S 3G9, Canada; [6]Division of Metabolism and Children's Research Center, University Children's Hospital Zürich, University of Zürich, CH-8032 Zurich, Switzerland; [7]Invitae Corp, San Francisco, CA 94103, USA; [8]Department of Human Genetics, McGill University, Montreal, QC H3A 0C7, Canada; [9]Department of Pediatrics and Inherited Metabolic Disorders, Charles University, First Faculty of Medicine and General University Hospital in Prague, Ke Karlovu 2, 12 08 Praha 2, Czech Republic
*Correspondence: fritz.roth@utoronto.ca
https://doi.org/10.1016/j.ajhg.2021.05.009.
© 2021 The Authors. This is an open access article under the CC BY license (http://creativecommons.org/licenses/by/4.0/).

supplementation with folinate can remediate symptoms for mild hyperhomocysteinemia cases.[25]

MTHFR is most widely known because of the common variant p.Ala222Val, which is carried by roughly half of all humans (global allele frequency 31%, gnomAD[26,27]). This variant, which corresponds to c.665C>T (sometimes noted as "c.677C>T" on the basis of outdated coding sequence annotation), decreases MTHFR's thermostability and affinity to flavin adenine dinucleotide (FAD) and yields reduced enzyme activity at low substrate availability.[28–32] The modestly increased plasma homocysteine levels associated with p.Ala222Val are readily ameliorated by increased dietary folate.[25] Although pregnant women who are homozygous for c.665C>T (p.Ala222Val) are advised to follow a high-folate diet to avoid neural-tube birth defects, this advice is commonly given to all pregnant women irrespective of p.Ala222Val status, and whether there are broader health effects of the p.Ala222Val variant remains controversial.[33] However, approximately one-third of rare or private variants in MTHFR occur *in cis* with p.Ala222Val, and the extent to which p.Ala222Val modulates the effects of other MTHFR variants or their responsiveness to folinate supplementation is unclear, further complicating MTHFR variant interpretation.

Here we applied multiplexed assays to generate an atlas of the impacts of MTHFR variants across environmental and genetic contexts.

Material and Methods

Overview of multiplexed measurements of MTHFR-variant effects

Here we provide an overview of the methods used for MTHFR variant effect mapping and follow it with additional detail.

Functionally testing all possible amino acid substitutions in a target protein demands a scalable assay. Here we took advantage of the fact that *S. cerevisiae* yeast strains lacking the MTHFR ortholog *MET13* normally cannot grow without supplemented methionine but are rescued by expression of human *MTHFR*.[30] Because yeast (unlike humans) can synthesize folate, we used an extension of this assay in which yeast *FOL3* is also deleted, enabling external control of intracellular folate via titration of folinate (5-formyl-tetrahydrofolate) in growth media.[34]

Using the coding sequence of the canonical splice isoform of *MTHFR* (Ensembl: ENST00000376590.9; GenBank: NM_005957.5), we produced mutagenized cDNA libraries from both reference ("wildtype," or WT) and p.Ala222Val variant templates. Each variant should be sufficiently well represented in its library to enable accurate measurement of variant frequency, but we also wanted to limit the number of variants per clone. We therefore generated multiple full-length cDNA libraries, each targeted by oligo-directed mutagenesis in a different region. MTHFR mutant libraries for each of four regions in each of the two genetic backgrounds were separately cloned *en masse* into yeast expression vectors and transformed into the assay strain described below.

Each of the pools of MTHFR-variant-expressing yeast strains was grown both with methionine supplementation (the non-selective condition) and without (selective condition), at each of four different concentrations of folinate (12.5, 25, 100, and 200 μg/mL).

We assessed variant effects by using the TileSeq approach, which involves extracting DNA corresponding to the target gene locus from pooled cells and sequencing segments of the target gene ("tiles") at a depth (typically >2M reads) sufficient for the "allele frequency" of each variant to be estimated and compared between the selected and unselected cell populations. An advantage of using short (~130 nt) tiles is that both strands can be sequenced on an economical short-read sequencing platform, which in turn enables greatly reduced base-calling error and accurate estimates of low (parts per million) allele frequencies. A disadvantage is that any given clone might carry additional unseen variations outside of the sequenced tile. However, one can robustly estimate the underlying frequencies of alleles by maintaining pool complexity that is sufficiently high for the average impact of additional unseen variation to be relatively constant for all variants. It has been previously shown that TileSeq can measure variant effects with an accuracy on par with that of approaches based on full-length clone sequencing.[10]

The use of regional mutagenesis in combination with TileSeq overcomes the challenges presented by longer proteins, in which it is difficult both to have a modest number of mutations per clone and to represent each variant at frequencies distinguishable from random errors arising from amplification and sequencing.

A functionality score and associated measurement error was derived for each variant (see supplemental methods A) in each folinate concentration and genetic background. Because mutagenesis and selection were carried out separately for each region, scores from each region were rescaled before they were combined into a single overall variant effect map (see supplemental methods A).

MTHFR assay strain and validation

The yeast strain (*MATα fol3Δ::KanMX met13Δ::KanMX his3Δ1 lys2Δ0 leu2Δ0 ura3Δ0*), MTHFR ORF clone (Ensembl: ENST00000376590.9; GenBank: NM_005957.5, corresponding to UniProt: P42898-1), and variant p.Ala222Val clone that we used for the complementation assay, as previously described,[34] were kindly provided by Dr. Jasper Rine. We subcloned WT and mutant MTHFR ORFs into a Gateway-compatible yeast expression vector pHYC-Dest2 (CEN/ARS-based, *ADH1* promoter, and *LEU2* marker) to enable large-scale complementation.

We validated our implementation of this system in low-throughput liquid growth assays (by using a Tecan microplate reader) and evaluated MTHFR WT, p.Ala222Val, and null controls at six different levels of folinate supplementation (0, 6.25, 12.5, 25, 50, and 100 μg/mL). The growth assay confirmed that human MTHFR complements loss of yeast *MET13* and that the p.Ala222Val variant displays a modest fitness defect, which increased in severity at diminished folinate concentrations (Figure S1A).

Mutagenized library construction

We used the POPCode (Precision Oligo-Pool Based Code Alteration) method to generate codon-randomized MTHFR variant libraries.[10] Because *MTHFR* (1971 bp) is longer than any gene previously mutagenized with POPCode, mutagenesis was performed regionally: the coding sequence was subdivided into four regions for the purpose of mutagenesis, such that four full-length MTHFR libraries in which each region has been targeted in turn for random codon mutagenesis were generated. In brief, 28–38 bp oligonucleotides, carrying centrally located NNK-degenerate codons, were designed along the entire length of the MTHFR ORF.

Oligos for each region were combined to produce four regional oligo pools. Uracilated full-length WT MTHFR (or p.Ala222Val MTHFR) was used as the template, and four separate annealing reactions (involving Kapa HiFi Uracil+ DNA polymerase [KapaBiosystems] and a mix of dNTP/dUTP) were set up with the regional oligos. Once the oligos had annealed, the gaps were filled in via KAPA HiFi Uracil+ DNA polymerase (Kapa Biosystems), and nicks were sealed with Taq DNA ligase (NEB). The original uracilated template was then degraded with Uracil-DNA-Glycosylase (UDG) (NEB), and the newly synthesized mutagenized strand was amplified through the use of primers containing attB sites. We then transferred the PCR product thus generated *en masse* into pDONR223 via Gateway BP reactions to generate Entry clones (we pooled 100,000 clones per library to maintain complexity).

Subsequently, we transferred the Entry libraries into a pHYC-Dest2 expression vector by using *en masse* Gateway LR reactions (we pooled ~300,000 clones to maintain complexity) to enable yeast expression. These regionally mutagenized expression libraries were then each transformed into the *S. cerevisiae met13fol3* strain. Then, in order to maintain high complexity, we pooled at least 300,000 clones to generate regional host libraries.

Multiplexed assay for MTHFR variant function

The underlying yeast-based functional complementation assay of MTHFR has previously been established by Marini and colleagues[34] for the evaluation of individual variants. Details are provided here for high-throughput complementation screening: in brief, for each region plasmids were extracted from two ~2.7×10^8 cell pellets of the *S. cerevisiae met13 fol3* regional transformant pools, yielding ~300,000 total transformants. These plasmid preparations were used for downstream tiling PCR. These two plasmid pools served as the two biological replicates for the non-selective condition.

We washed approximately 3×10^8 cells from the regional transformant pools three times to eliminate any residual methionine and subsequently inoculated the cells into selective media. We used synthetic complete (SC) media without leucine (USBiological) (to ensure plasmid retention) as the nonselective growth media. For selective media, we used synthetic defined (SD) media lacking methionine; this was made with a yeast nitrogen base lacking vitamins or amino acids (US Biological). To relieve auxotrophies in the mutant strain, we supplemented the selective media with standard concentrations of histidine, lysine, uracil, and all other vitamins except folinic acid. We then added folinate to the selective media at four different concentrations: 12.5, 25, 100, and 200 μg/mL.

For each of the eight pooled complementation assays (four folinate concentrations for each of two genetic backgrounds), two replicates of cells in selective media were allowed to grow to full density (5–6 doublings) with shaking at 30°C. 2.7×10^8 cells were harvested from each replicate, and plasmid DNA was extracted from these cells. This DNA was subsequently used as a template for tiling PCR. In parallel, the *met13 fol3* strain was similarly transformed with the WT ORF, and this control strain was grown in 10 mL nonselective and selective media alongside the regional pools to serve as WT controls.

Sequencing and scoring strategy

We used the previously described TileSeq strategy for reading out the effects of selection.[10] MTHFR was divided into four regions for separate mutagenesis and selection; each region was subdivided into tiles for sequencing-based measurement of variant frequencies (for a total of 19 tiles). Each tile was short enough to enable full-length sequencing of both strands to achieve low-base-calling error and thus enable accurate measurement of extremely low (parts-per-million) variant frequencies. Each region consisted of four to six tiles (R1: four tiles, R2: four tiles, R3: five tiles and R4: six tiles). For each of the plasmid libraries from non-selective and selective pools, tiling PCR was performed as previously described.[10,12] In brief, each tile was amplified with primers carrying a binding site for Illumina sequencing adapters. These first-step amplicons were then indexed with Illumina sequencing adapters in the second-step PCR. Paired-end sequencing was performed with an Illumina NextSeq 500 via a NextSeq 500/550 High Output Kit v2 on every tile from all conditions and from the WT control. The relative abundance of each library was adjusted so that there was a sequencing depth of ~2 million reads for each tile.

TileSeq read data were processed as previously described.[10,12] In brief, conditions were demultiplexed with Illumina bcl2fastq. Variant allele frequencies in each condition were calculated via the tileseq_package, which uses bowtie2[35] to align each read pair to the template and call mutations when both reads for each tile agree on its presence. Across all tiles, 99.1% of the reads could be mapped to the template, and 87.5% of read-pairs agreed on all variants called. Variants with disagreement within a read pair were treated as wildtype. Counting and sequencing-depth normalization were performed with custom software (tileseq_package v1.5, see code availability section). This yielded variant frequency data for each condition and replicate. Extrapolating from the mutations observed in each tile, we estimated an average of 0.93, 0.86, 0.8, and 0.71 codon changes per clone for regions 1–4, respectively, and also modeled the distribution of numbers of codon changes per clone expected under a Poisson distribution (Figure S2). Raw fitness values were calculated from the sequencing reads as previously descibed[10,12] with the tileseqMave pipeline (see "data and code availability"). Here, we updated tileseqMave to allow scores from each region to be separately rescaled, such that the scores of zero and one were defined by the respective modes of nonsense and synonymous variants. See supplemental methods A for analysis details.

Structure modeling and determination of binding-site residues

To obtain a structural model of MTHFR, including all relevant binding sites, we used OpenPyMol to generate a structural alignment between the human MTHFR structure obtained from Froese and colleagues[36] (PDB:6FCX) and its *E. coli* ortholog bound to the substrate proxy LY309887 by Pejchal and colleagues[37] (PDB:2FMN). We then extracted lists of all residues within 5Å from LY309887, from FAD, and from SAH, as well as from the dimerization interface.

Modeling dependence of variant functionality on folinate and p.Ala222Val

A full description of the modeling approach can be found in supplemental methods B. In brief, the response of each variant to folinate supplementation was modeled via a parsimonious linear function that expresses the functionality score at a given concentration in terms of a base functionality and a folinate response parameter. The maximum likelihood model for each variant was determined and compared with the corresponding null model, which assumes no supplementation response. This null-model

likelihood allows for the determination of a log likelihood ratio (LLR) expressing how much more likely the supplementation response model is compared to the null model. We used a similar approach to model the genetic interaction between a variant and p.Ala222Val. To obtain log likelihood ratio of genetic interaction (LLRg) values, we first expressed the expected double-mutant functionality score via polynomial regression between the functionality scores in the WT and p.Ala222Val backgrounds. We then derived folinate-independent and folinate-dependent models of the score expected for the combination of each variant with p.Ala222Val and subsequently estimated maximum likelihood and LLRg as we did for LLR.

Identification of p.Ala222Val suppressor variants

To identify genetic suppressors of p.Ala222Val, we first established the distribution of synonymous variants in the p.Ala222Val background and calculated their 95th percentiles in each tested folinate concentration. These correspond to the functionality score threshold above which a variant is likely to exhibit p.Ala222Val-like functionality less than 5% of the time (i.e., the threshold achieving an empirical $p < .05$ in a one-tailed test). We then searched for variants that achieved a good fit in the genetic interaction model (log likelihood > -8) and had a greater than 95% posterior probability of a positive genetic interaction (either folinate-dependent or -independent). We then checked which of these variants achieved double-mutant functionality scores that fell above the aforementioned synonymous threshold at low folinate (12.5 μg/mL), high folinate (200 μg/mL), or all folinate concentrations.

Molecular dynamics simulation of the FAD binding site

We used Amber Modeler 9.24 to perform molecular-dynamics simulations on the structural model described above. The distances between FAD, Trp165, and the active site for each simulation frame were calculated and analyzed with custom R code (see "data and code availability" section). A Markov Model of the detailed molecular interaction was also created with custom R code. A detailed description of this analysis can be found in supplemental methods C.

Identification of hypercomplementing variants

To determine the set of hypercomplementing variants, we first determined the distribution of base functionality scores of all measured synonymous variants in the WT background. Then, for all missense variants with base functionality greater than 1 (= WT-like), we used Welch's t test (incorporating each variant's mean, standard deviation and number of replicates) to determine whether their functionality was significantly greater than the distribution of the synonymous variant. We used Benjamini & Hochberg's FDR correction method[38] to correct p values for multiple-hypothesis testing and selected variants that achieved FDR < 5%.

In vitro assay for MTHFR activity

To test whether variants had an effect on MTHFR activity, and whether these effects were altered by FAD or S-adenosylmethionine (SAM) supplementation, we used an *in vitro* assay as previously described;[39,40] we used 293T cells (ATCC: CRL-3216) modified by genetic editing to harbor MTHFR knock-out (KO) or specific variants.[41] In MTHFR-KO 293T cells, specific activity was measured after transfection with empty vector (pcDNA-C-Flag-LIC), vector harboring WT MTHFR (corresponding to GenBank: NM_005957.5), or variant MTHFR constructs generated by site-directed mutagenesis.[31] The assay was performed in 50 mM potassium phosphate buffer (pH 6.6) under saturating substrate concentrations (100 μM methyleneTHF; 200 μM NADPH) in the absence or presence of 75 μM FAD (for FAD responsiveness studies) or in the presence of 75 μM FAD after preincubation for 5 min at 37°C with purified SAM[42] (for SAM inhibition studies). The K_i for SAM was estimated from a plot of inhibitor versus response and a four-parameter curve fit (GraphPad Prism v8.00). For heat-treatment studies, samples were incubated at 46°C for 5 min before the assay, either without FAD supplementation or with 75 μM FAD supplemented either before or after heat treatment, as described.[40]

Benchmark reference variant sets

To evaluate the ability of map scores to predict variant pathogenicity (and ultimately individual phenotypes), we needed to establish positive and negative variant reference sets. A full description of the process can be found in supplemental methods D. In brief, for the positive reference set, hyperhomocysteinemia case genotypes and phenotypes were assembled largely from previous publications.[13,43] Variants that were seen more often in early-onset than late-onset cases were included in the "early-onset positive reference set" (comprising 30 variants), whereas variants that were more often observed in late-onset cases than early-onset cases were included in the "late-onset reference set" (comprising 40 variants). Ties were excluded.

To obtain a random reference variant set, we accessed gnomAD[26,27] (a collection of genotypes meant to be comprised primarily of unaffected individuals) and filtered for missense MTHFR variants that fulfilled one of the two following criteria to enrich for variants likely to be benign: (i) the global minor-allele frequency is above 1 in 10,000; or (ii) at least one homozygous case has been observed. Full details of the filtering steps can be found in supplemental methods E.

Determining a log-likelihood ratio of pathogenicity for each variant

The evaluation against reference sets showed that functionality scores in the catalytic and regulatory domains were not on a comparable scale in terms of predicting variant pathogenicity. It is perhaps not surprising that the extent to which cells rely on each of MTHFR's functions differs between humans and yeast. Separately for each of these domains, we implemented a transformation function to represent variant effects in terms of the strength of evidence for and against pathogenicity, that is, a log-likelihood ratio of pathogenicity (LLR_P). To this end, we used the distributions of functionality scores for the positive and random reference sets for both the best-performing experimental map (p.Ala222Val background at 25 μg/mL folinate) and for the linear model with best-performing parameters (WT background at 120 μg/mL folinate; see Figure 6A) to construct likelihood ratios, while considering catalytic and regulatory domains separately. The details of the underlying calculations can be found in supplemental methods F.

Assigning personalized MTHFR functionality scores to diploid genotypes

As a positive reference set of diploid genotypes, we extracted from our literature-curated table cases that featured at least one missense variant and provided information both about age of onset and p.Ala222Val status. As a random reference set, we

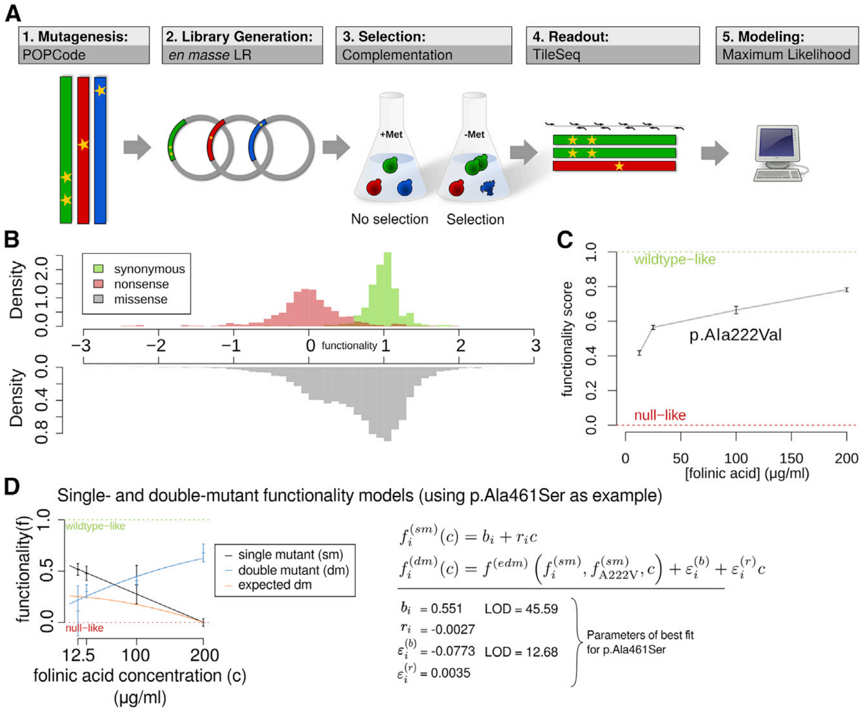

Figure 1. Overview of variant-effect-map generation and validation
(A) The variant-effect mapping process.
(B) Distribution of scores for synonymous, nonsense, and missense variants in the WT background at 12.5 μg/mL folinate supplementation.
(C) The behavior of p.Ala222Val across folinate concentrations. Error bars correspond to SEM.
(D) Folinate dependence is shown for the variant p.Ala461Ser in both WT (single mutant, black) and p.Ala222Val (double mutant, blue) backgrounds. Data points are colored according to genetic background, and error bars represent SEM. Lines show best fitting models (parameters at right). The orange line indicates the double-mutant behavior expected on the basis of the polynomial regression model. The red and green dotted lines represent typical null-like and WT-like scores, respectively. Single- and double-mutant modeling equations are shown at the bottom.

obtained individuals from 1000 Genomes Phase III for which phased genotypes at the p.Ala222Val locus were available and selected individuals with at least one missense variant.

To provide models of the relationship between diploid genotype and phenotype, we defined four different models of increasing complexity. All models involved converting functionality scores for each allele into log-likelihood ratios of pathogenicity (as above) by using the appropriate transformation depending on whether the variant fell within the catalytic or regulatory region as defined above. To reflect the recessive nature of MTHFR deficiency, we assigned a diploid genotype to the lowest (least likely to be pathogenic) LLR_p of the two alleles. The first model (M_1) interprets variants as if they were in the WT background, whereas the second model (M_2) accounts for genetic interaction. The third model (M_3) accounts for an additional *in cis* common variant, p.Glu429Ala (E429A) and its genetic interaction with p.Ala222Val. A formal description of the models can be found in Supplemental Methods G.

Results

Applying a scalable functional assay to MTHFR missense variants

Functionally testing all possible amino acid substitutions demands a scalable assay, which was offered by the fact that human *MTHFR* expression allows *S. cerevisiae* lacking the *MTHFR* ortholog *MET13* to grow without supplemented methionine.[30] Because yeast (unlike humans) can synthesize folate, we used a variant of this assay[34] enabling external control of intracellular folate via titration of folinate in growth media. After validating the system (Figure S1A), we produced mutagenized cDNA libraries from both reference ("wild-type" ; WT) and p.Ala222Val variant templates. We generated multiple full-length cDNA libraries, each targeted by oligo-directed mutagenesis in one of four regions. (See material and methods for details).

Mutagenized libraries for each region in both WT and p.Ala222Val backgrounds were cloned *en masse* into yeast expression vectors and transformed into the assay strain (Figure 1A). Each of the resulting strain pools was grown in selective and non-selective conditions at each of four different concentrations of folinate (12.5, 25, 100, and 200 μg/mL). Relative abundance of variants in each post-growth pool was assessed via ~2 million sequencing reads from the mutagenized region of each library. (See material and methods for details).

A functionality score and associated measurement error was derived for each variant in each folinate concentration and genetic background. After filtering out scores for amino acid substitutions that were poorly measured (e.g., because they were not well represented in the non-selective library), we obtained scores for 91% (±0.3%) and 88% (±0.8%) of the 13,776 possible mutational outcomes (amino acid changes, nonsense or synonymous) in WT and p.Ala222Val-background MTHFR, respectively (scores were averaged across folinate conditions; see Table S1 for details). Functionality scores were rescaled such that a score of 0 corresponds to a complete loss of function and a score of 1 corresponds to WT-like growth, thus yielding an atlas of 98,336 MTHFR-missense-variant effect scores, collected across eight different environmental/genetic contexts.

Functionality scores of synonymous and nonsense variants were generally well separated from one another, and the distribution of missense-variant functionality scores appeared to be bimodal (Figure 1B), indicating that many MTHFR missense variants have either all or no function

and that a minority have intermediate effects (see material and methods for details).

The known hypomorphic p.Ala222Val variant was scored at 41.8% of WT functionality in the lowest folinate condition; this score increased with folinate concentration (Figure 1C), consistent with previous reports that p.Ala222Val has reduced WT enzymatic activity and is remediable by folinate supplementation.[28,29,44] A large fraction of other variants were less functional in the p.Ala222Val background than in the WT background. Among 11,902 missense variants measured in the maps, 5,836 were significantly less functional in the p.Ala222Val background (Welch test with FDR $q < 0.05$) in at least one tested folinate concentration, and 1,203 variants were significantly less functional in all tested concentrations. Conversely, only 20 variants were significantly more functional in p.Ala222Val in all concentrations, and 372 variants were more functional in at least one concentration (a full list of affected variants is in Table S2). We compared our atlas to previously published low-throughput measurements of enzyme activity for seven variants in WT and p.Ala222Val backgrounds.[45] We found a high correlation with our maps, particularly at high (200 μg/mL) folinate levels (Spearman's rho = 0.93; Figure S1B).

Modeling "base functionality," folinate response, and genetic interaction

For each variant, we used a linear model to infer a "base functionality" (folinate-independent) score (*b*) and a folinate responsiveness score (r) to obtain well-fit models for 78% of variants (Figure S3A). An example fit is shown in Figure 1D (black line). We also estimated the likelihood of each variant's model relative to a simpler control model that did not capture folinate responsiveness. For example, the four measurements of p.Ala222Val itself from the four WT maps was 6×10^{14} times more likely under the linear model (in which functionality score increased by 0.0016 for every μg/mL of folinate supplementation) than under the best-fitting control model. As another example, our data for p.Trp165Cys (discussed further below) are 5×10^{39} times more likely under the linear model than under the control model, and the functionality score increases 0.0027 per μg/mL folinate. Of the 12,620 MTHFR variants successfully modeled, we could confidently identify 895 (7%) as being folinate-responsive (Table S2). (See Supplemental Methods for details.)

To capture genetic interactions between each variant and the common p.Ala222Val polymorphism (i.e., cases where the phenotypic effects of a combination of two variants deviate from expectation), we initially modeled the expected behavior of double mutants via polynomial regression by taking functionality scores for the two corresponding single mutant scores as inputs (Figure S3B, Figure 1D, orange line). We then modeled the genetic interaction strength (ε) for each variant as the sum of folinate-independent (ε_b) and folinate-dependent (ε_r) genetic interaction terms. We identified all variants that confidently departed from simpler models missing all genetic interaction or only folinate-dependent genetic interaction terms. Of 10,136 variants modeled, we found that 3,359 (33.2%) genetically interact with p.Ala222Val; 2,839 (28%) have a folinate-independent and 521 (5.1%) have a folinate-dependent genetic interaction (Table S2). (See supplemental methods for details.)

The best-fit parameters from this model allow us to describe MTHFR variants in terms of four characteristics: (1) overall functionality of the variant, (2) impact of folinate supplementation on variant function, and each variant's (3) folinate-independent and (4) folinate-dependent genetic interactions with the common p.Ala222Val background. Figure 2 shows a variant effect map for each of these characteristics for one segment of MTHFR (the full set of maps over the entire protein length is shown in Figure S4).

The structural context of variant effects

Eukaryotic MTHFR has two domains connected by a short linker sequence.[36] An N-terminal catalytic domain in a TIM-barrel fold holds FAD as a cofactor at the active site, which is located at the top of the barrel. The C-terminal regulatory domain includes a homodimerization interface and a single binding site that can contain either S-adenosylmethionine (SAM) or S-adenosylhomocysteine (SAH). SAM induces a conformational change that propagates through the MTHFR linker domain to inactivate the catalytic domain.[36] SAH binds competitively and blocks SAM inhibition. A short, disordered, serine-rich region at the N terminus harbors many phosphorylation sites that can yield increased sensitivity to SAM inhibition.[36]

Substantially reduced functionality of variants in the catalytic relative to the regulatory domain was visually evident in every map ($p < 2.2 \times 10^{-16}$; Mann-Whitney $U = 1 \times 10^7$). This phenomenon was also observed ($p = 0.015$; Mann-Whitney $U = 22$) for the subset of variants present in a random reference cohort (see material and methods). A greater general impact for catalytic residues is consistent with a significantly lower median allele frequency of random reference cohort variants in the catalytic domain ($p = 0.026$; Mann-Whitney $U = 45$) and with the observation that 12 out of the 13 missense variants observed to be homozygous in the random reference cohort were located in the regulatory domain.

Residues in the protein core and at binding interfaces were expected to be more sensitive to mutation than non-interfacial residues at the surface. Indeed, for amino acids with <20% solvent-accessible surface area (ASA), the median score of substitutions was lower than that of amino acids with >50% ASA, both in the regulatory domain and the catalytic domain (Δmedian = 0.151 and Δmedian = 0.545 and $p = 1.6 \times 10^{-8}$ and $p = 5.3 \times 10^{-12}$, respectively, by Mann-Whitney U test; Figure 3A). Similarly, for amino acids within 5Å of the substrate (5,10-methylenetetrahydrofolate) or cofactor (FAD) binding sites in the catalytic domain, substitutions showed more severe functionality defects than did those in surface

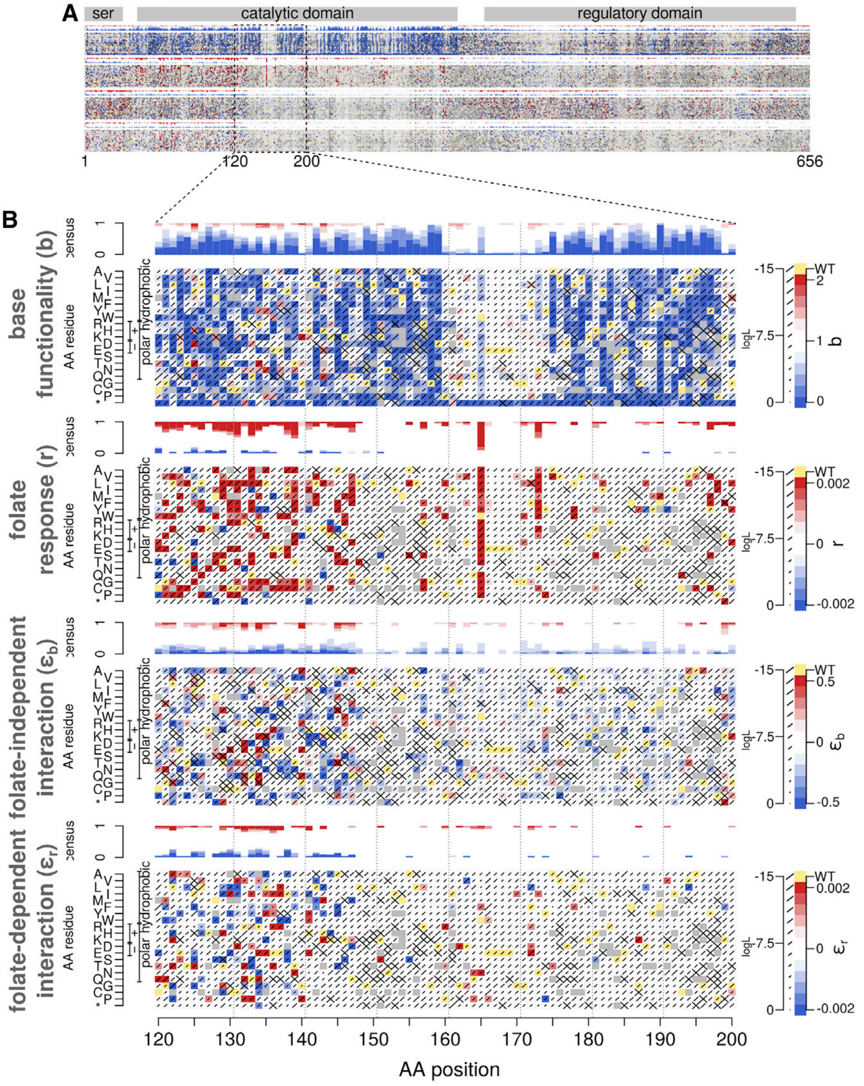

Figure 2. MTHFR variant effect maps for base functionality, folinate response, and both folinate-independent and -dependent genetic interactions with p.Ala222Val

(A) Preview of full-sized maps.

(B) Expanded view of positions 120–200. In descending order, maps represent base functionality (functional impact in WT background at low folinate levels); functionality response to folinate supplementation; and folinate-independent; and -dependent genetic interactions with p.Ala222Val. For each map, the x axis shows MTHFR amino acid position, and the y axis corresponds to possible amino acid changes. Diagonal bar sizes indicate the estimated error in the corresponding score. Gray squares indicate missing data, and yellow squares indicate the WT amino acid at each position.

residues (Δmedian = 0.518 and Δmedian = 0.59 and p = 3.3×10^{-4} and p = 1.4×10^{-4}, respectively, by Mann-Whitney U test). Interestingly however, residues within 5Å of bound SAH (binding at the regulatory domain) did not exhibit lower scores than surface residues. This could indicate that SAM suppression is not strictly required for cell growth under the conditions of our assay. Similarly, the scores for residues located at the dimerization interface were no lower than scores for residues located at the remaining surface of the regulatory domain, which matches previous observations that dimerization is not strictly necessary for MTHFR function.[36]

The structural context of environmental and genetic interactions

We hypothesized that folinate-responsiveness of variant effects would be enriched within the catalytic domain of MTHFR, and this was indeed the case (OR = 3.32 and $p < 2.2 \times 10^{-16}$, Fisher's exact test). Folinate responsiveness was most evident in the half of the catalytic domain that is spatially proximal to the regulatory domain (Figure 3B), including a prominent hotspot around position 325 in structural proximity of the folate polyglutamyl tail, and at position 165 within a disordered loop (explored further below). Additional hotspots of folinate responsiveness were also seen at other (ordered) loop structures near amino acid positions 200, 230, and 270, which together form a structural cluster near the FAD binding site.

A direct comparison of base functionality and responsiveness scores (Figure 3C) for the highest-quality models (log likelihood > −6) revealed that hypomorphic variants are the most likely to be responsive to folinate supplementation, whereas null-like and WT-like variants were least likely to be responsive.

Assessing the tendency of different structural regions to exhibit genetic interaction, we found the serine-rich region, along with N-terminal halves of both the catalytic and regulatory domains, to be enriched for both folinate-dependent and -independent genetic interactions. An additional hotspot of folinate-independent interactions was seen from positions 560–610, and enrichment for folinate-dependent interactions was observed at positions 320–330 (Figure S3C).

To determine whether there are variants that can suppress the hypomorphic effects of the p.Ala222Val allele, we sought well-modeled variants with confident positive genetic interactions, where the double-mutant functionality was significantly greater than that of p.Ala222Val alone (See material and methods for details). Of 10,136 well modeled variants, 636 (6.3%) suppressed p.Ala222Val. These could be subdivided into 232 folinate-independent suppressors, 385 variants that only suppressed p.Ala222Val

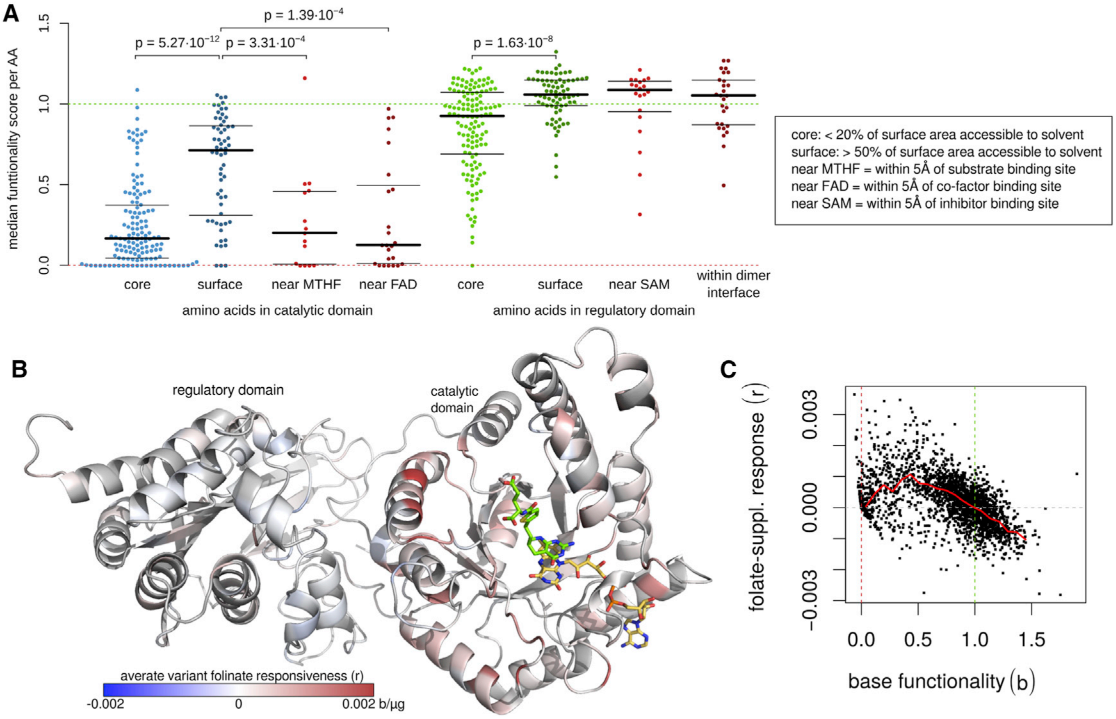

Figure 3. Modeling folinate supplementation response and genetic interaction

(A) Median functionality scores of variants at amino acid positions with the following properties: (1) below 20% accessible surface area (ASA) in the catalytic domain; (2) above 50% ASA in the catalytic domain; (3) within 5Å of bound MTHF; (4) within 5Å of bound FAD; (5) below 20% ASA in the regulatory domain; (6) above 50% ASA in the regulatory domain; (7) within 5Å of bound SAH; (8) more than 20% ASA reduction when in dimer form. p values were calculated by Mann-Whitney U test. Thick and thin bars correspond to median, upper, and lower quartiles, respectively.

(B) Structure visualization of MTHFR with residues colored according to the average intensity of significant folinate-responsive variants at each position. Red positions indicate a positive folinate response, blue positions indicate a negative folinate response, and white positions indicate no response. FAD and folinate are shown in yellow and green wireframe representations.

(C) Scatterplots comparing the maximum-likelihood model base functionality (b_i) and folinate-supplementation response parameters for each variant. The red line shows a running median across the x axis (interval size 0.1); the dotted dark red, dark green, and gray lines indicate null-like functionality, WT-like functionality, and zero response, respectively.

at low folinate levels, and 19 variants that only suppressed at high folinate levels. Figure S3D shows the strongest suppressor in each category. Considering only substitutions that can result from a single-nucleotide substitution (and are therefore more likely to be observed in human populations), we found 70 folinate-independent, 152 low-folinate, and 10 high-folinate suppressors. (See Table S2 for full list).

A disordered loop might serve to tether the FAD cofactor

A crystal structure for human MTHFR had revealed an unconserved, disordered loop, stretching from residues 159 through 174[36] within the catalytic domain, to "overhang" the active site (Figure 4A). Loop residues, in stark contrast with the surrounding catalytic domain, were generally tolerant to mutation in our atlas (Figure 4B). Although surrounded by tolerant loop positions, the tryptophan at position 165 showed strong and folinate-dependent functionality defects for most amino acid replacements (Figure 4B). Given a previously observed relationship between folinate levels and retention of the FAD cofactor in the p.Ala222Val variant,[28] as well as our atlas-derived observation of folinate-dependent functionality impacts in a loop proximal to the FAD binding site, we wondered whether Trp165 could be important for FAD retention in the absence of the MTHFR's folinic-acid derived substrate.

To explore the role of this loop more closely, we performed molecular-dynamics simulations for the WT protein, for the polar substitution p.Trp165Gln, and for the aromatic substitution p.Trp165Tyr. Under all initial conditions examined, Trp165 tended to associate with the flavin group of the FAD cofactor, frequently with orientations suggesting a π-stack between aromatic rings. Consistent with the functionally abnormal map scores for p.Trp165Gln, the residue's alpha carbon wandered significantly more often from the FAD flavin than it did either in the WT structure ($p = 0.0001$, Mann-Whitney U test) (Figure 4C) or with aromatic substitution p.Trp165Tyr ($p = 0.019$, Mann-Whitney U test). (See material and methods for details).

To test the hypothesis that the loop, via Trp165 binding, serves to retain FAD in the active site, we also evaluated the

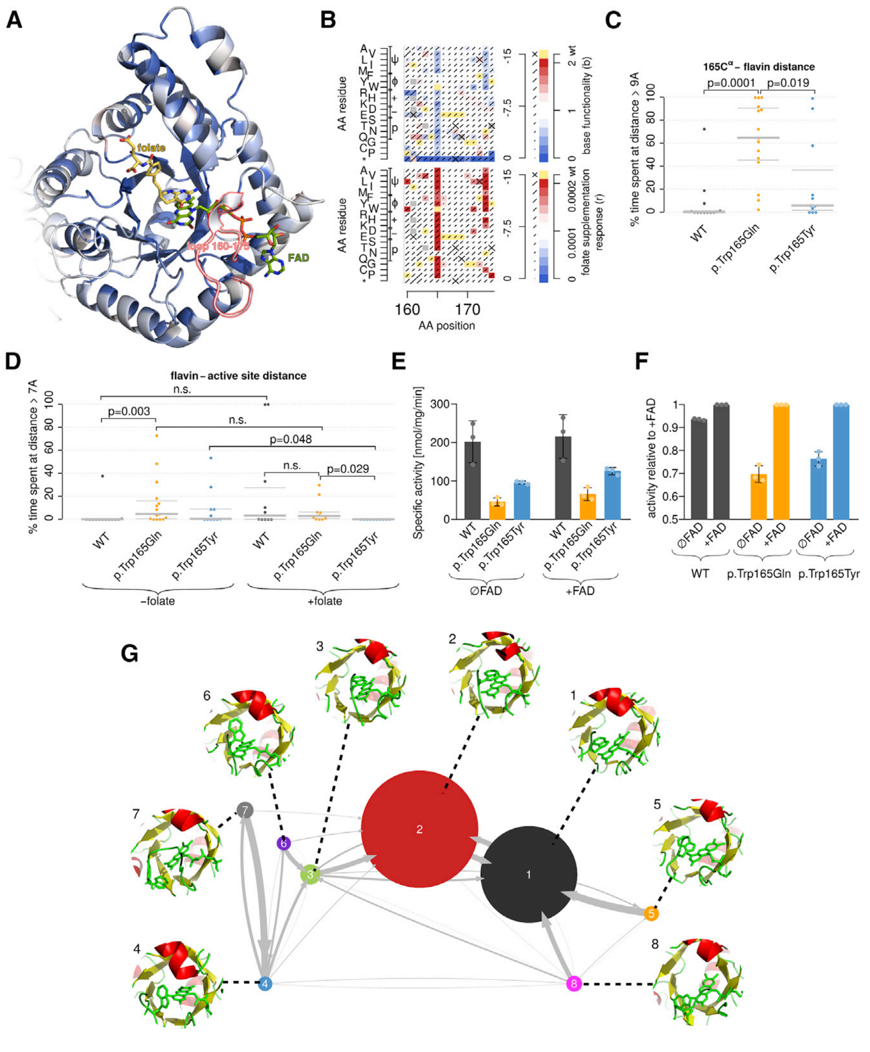

Figure 4. Structural context and molecular-dynamics simulations
(A) Structural model of MTHFR's catalytic domain; coloring is according to median base functionality of substitutions at each position. Wireframe models of the folate-analog (yellow) and FAD (green) are shown, along with the disordered loop (red).
(B) Heatmaps of base functionality and folinate-supplementation response for disordered-loop residues; colors are as in Figure 3.
(C) Fraction of MD simulation time spent where the residue 165 alpha carbon was ≤9Å from the FAD flavin group. p values are shown for Mann-Whitney U-tests.
(D) Fraction of simulation time spent in states where the FAD flavin group was ≤ 7Å from the active site center. p values are shown for Mann-Whitney U-tests.
(E) Specific enzyme activity of WT and Trp165 variants in the presence and absence of FAD. Error bars show standard deviation.
(F) Enzyme activity of WT and Trp165 variants relative to enzyme activity of the same variant in the presence of FAD. Error bars show standard deviation.
(G) A state space and transition model of binding modes between Trp165 and the FAD flavin group. Circle size represents time spent in each state, and arrow width represents transition probability.

movements of FAD in the same set of simulations. Although a > 7Å dislocation of FAD away from active site was numerically more common for the aromatic p.Trp165Tyr substitution, the difference was not significant ($p = 0.15$, Mann-Whitney U test). However, FAD dislocation was significantly more common for p.Trp165Gln than for WT ($p = 0.003$, Mann-Whitney U test) (Figure 4D; see material and methods for details).

Because p.Trp165Gln and other non-aromatic substitutions of Trp165 exhibited a strong impact only under low-folate conditions, we wondered whether FAD dislocation is rescued by the presence of folate. Repeating our simulations in the presence of the THF-analog LY309887, we did observe numerically less FAD dislocation for both p.Trp165Gln and p.Trp165Tyr; there was a significant reduction for the latter ($p = 0.029$, Mann-Whitney U test).

We next wished to confirm the effect of these mutants on *in vitro* enzyme activity (see material and methods). Consistent with our simulations, the activity assay showed that both p.Trp165Gln and p.Trp165Tyr have reduced enzyme activity compared to that of WT; p.Trp165Tyr was less severely impacted (Figure 4E). In comparisons of *in vitro* activity in the presence and absence of FAD, both variants appeared to be more responsive to FAD supplementation than was the WT, consistent with diminished retention of endogenous FAD (Figure 4F).

We also wished to examine whether these variants affected protein stability. We assessed activity for specific MTHFR variants after heat treatment before, after, and without FAD supplementation. For the p.Ala222Val control, enzyme activity was completely lost and did not recover unless FAD was supplemented before heat denaturation, as expected.[39] By contrast, the Trp165 variants, like WT: (1) retained some activity after heat treatment even without FAD supplementation, (2) retained more activity when FAD was supplemented after heat treatment, and (3) were completely protected by the addition of FAD before heat treatment (Figure S5). This suggests that Trp165 variants, consistent with their position in a flexible loop, do not reduce protein stability overall. However, in agreement with our simulation result, these results also suggest that Trp165 variants affect FAD binding and that there is a greater impact for the non-aromatic p.Trp165Gln substitution.

To further explore the interaction between Trp165 and FAD, we examined the MD simulations in more detail.

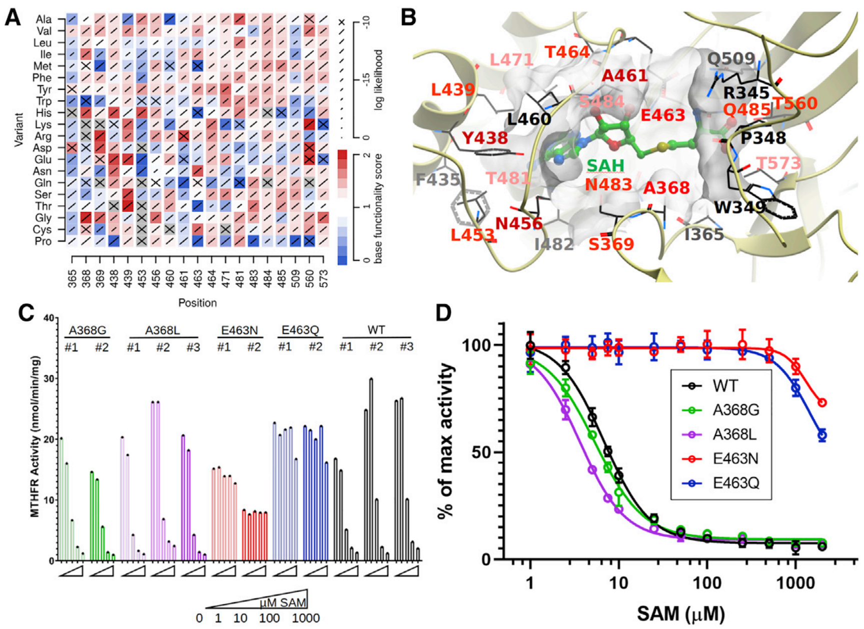

Figure 5. Hypercomplementation and loss of SAM suppression

(A) Variant effect scores for residues within 5Å of the SAM/SAH binding site, indicating hypomorphic (blue) and hypercomplementing (red) variants.

(B) Left: positions of the same residues in the MTHFR 3D structure relative to bound SAH.

(C and D) Enzymatic activity of p.Ala368Gly and p.Glu463Asn when expressed in HEK293 cells. (C) Absolute activity at different concentrations of SAM and (D) relative activity in response to increasing concentrations of SAM are shown. Error bars show standard deviation.

We spatially clustered structures from all time points of the WT simulations on the basis of the relative position and orientation of the aromatic rings of FAD and Trp165. This identified eight distinct binding modes (see material and methods). We also extracted transition rates to create a state transition model (Figure 4G), showing that all observed states "fed into" two dominant modes, together covering 96% of time points. In both dominant modes, a π-stacking interaction between Trp165 and the flavin group is apparent.

Together, our results indicate that this disordered loop, in the absence of folate and via direct π-stacking interaction between Trp165 and FAD's flavin group, aids in the retention of FAD. This role appears to be partially rescuable by other aromatic residues at position 165. In the presence of folate, Trp165 does not seem to be required for FAD retention. This is most likely because folate itself, via π-stacking interactions between the FAD flavin and the folate pteroyl group, secures FAD in place.

Hypercomplementation points to loss of SAM suppression

We identified 896 variants that provided growth rescue beyond that of WT MTHFR (FDR < 5%; see material and methods), a phenomenon previously described as "hypercomplementation."[10,12] Both visual inspection of the overall functionality map and statistical analysis showed that the regulatory domain was enriched for hypercomplementation (OR = 3.07; $p = 2.2 \times 10^{-16}$, Fisher's exact test; see Figures S4 and S6A).

Hypercomplementation could arise from disruption of the regulatory domain's ability to suppress MTHFR upon SAM binding, thus rendering it constitutively active. Hypothesizing that amino acid changes near the SAM binding site are more likely to disrupt this interface, we found that residues whose surface areas are more than 60% buried by SAM are more likely to hypercomplement than other regulatory domain residues (Fisher's exact test: OR = 1.69, $p = 6.3 \times 10^{-4}$, Figure S6A). Similarly, hypothesizing that variants reducing MTHFR's ability to distinguish SAM from SAH would be near the SAM methyl group, we found 16 of the 20 amino acids within 5Å of the methyl group to have multiple hypercomplementing variants (Figures 5A and 5B). These included Ala368, previously modeled to clash sterically with the SAM methyl group, and Glu463, previously predicted to be required for SAM binding.[36] At these two positions, the variants p.Ala368Gly and p.Glu463Asn showed particularly strong hypercomplementation phenotypes (Figure 5A).

To test whether p.Ala368Gly and p.Glu463Asn diminish SAM suppression, we used an established human cell-based assay[36,39,40] to examine the forward MTHFR reaction activity for these and other variants at these amino acid positions (Figures 5C and 5D and Figure S6). Activity assays of overexpressed proteins in MTHFR-KO HEK293 cells (Figure S6) and of endogenous proteins after genetic editing (Figures 5C and 5D) showed each variant to have WT-like maximum activity (Figure 5C and Figure S6B). Although variants at Ala368 were suppressible by increasing levels of SAM in the same manner as the controls (Figure 5D and Figure S6C), variants at Glu463 showed a loss of SAM suppressibility (Figure 5D and Figure S6D).

MTHFR variant effect maps correlate with variant pathogenicity

To initially assess the potential value of our atlas for clinical variant interpretation, we examined MTHFR variants previously observed in clinical sequencing by Invitae. Although phenotypes are not available for sequenced individuals, it is known which gene panel the physician requested for sequencing. Disease-specific panels (homocystinuria, fatty-acid oxidation defects, and neurometabolic disease) yielded variants with significantly lower map

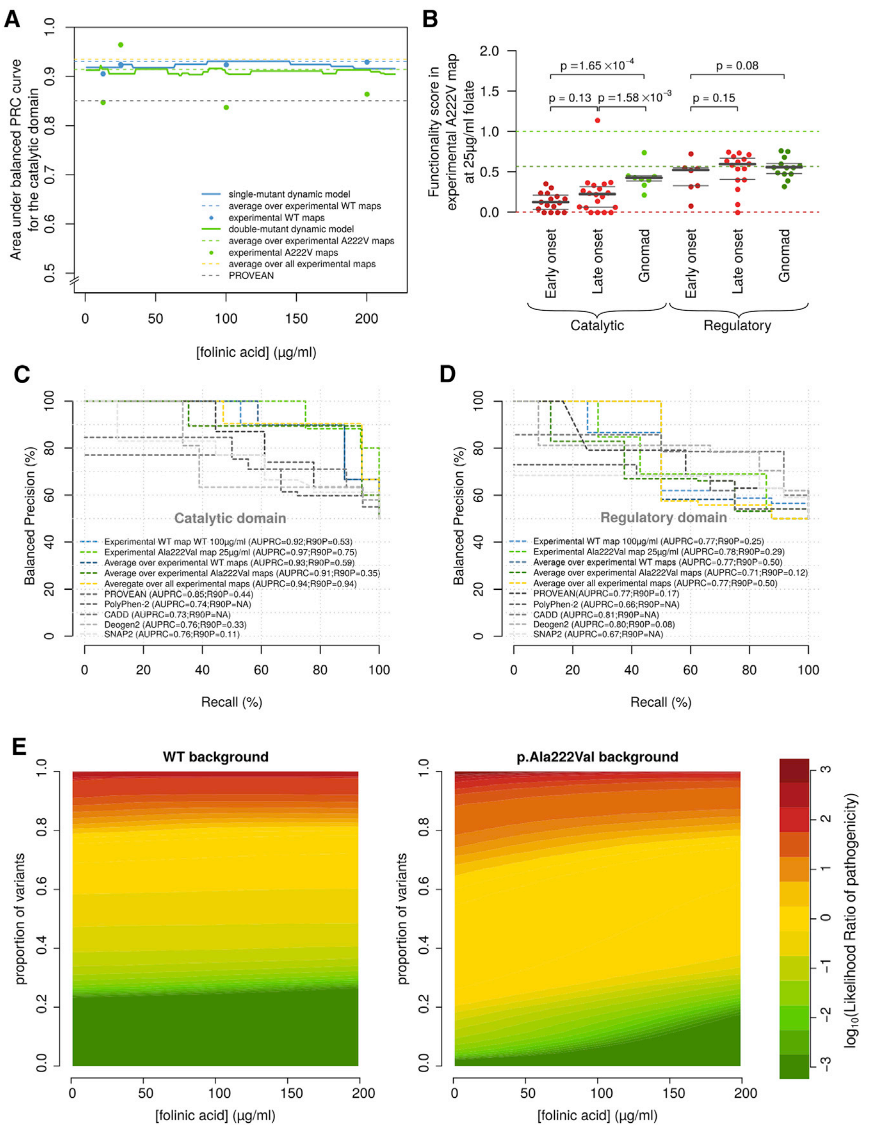

Figure 6. Evaluation of maps against pathogenic and random reference variant sets

(A) Area under the balanced precision-recall-curve (AUBPRC) for all experimentally measured maps and derived models is shown against (actual or virtual) folinate concentrations.

(B) Comparison of functionality scores in the p.Ala222Val background and 25 μg/mL folinate. Light green, dark green, and red dashed lines correspond to WT-like, p.Ala222Val-background-like, and null-like scores, respectively. Bold and light gray lines show medians and quartiles, respectively. p values correspond to Mann-Whitney U tests.

(C and D) Individual precision-recall curves of selected maps and models, as well as computational predictors PolyPhen-2, PROVEAN, CADD, Deogen2 and SNAP2 .

(E) Distribution of log-likelihood ratios of pathogenicity across folinate concentrations in both backgrounds.

scores (Figure S7A; Mann-Whitney U test, $p = 0.031$) than did non-specific panels (e.g., carrier and exome screens; see supplemental methods for details of statistical methods).

To enable assessment of the atlas, we established a benchmark comprised of a literature-curated positive reference set of variants that appeared most often in individuals with early-onset homocystinuria (see material and methods) and the above-described random reference set of variants found in the general population. We evaluated the area under the balanced precision recall curve (AUBPRC; see supplemental methods H) for the following: each individual map, maps derived from the mean of WT and the mean of p.Ala222Val scores, and a single combined map derived from an average of all maps, as well as "virtual maps" with interpolated scores from 0 to 200 μg/mL folinate (Figure 6A).

Scores tended, as expected, to be lower for positive than for random set variants but were visibly more so in the catalytic than in the regulatory domain (Figure 6B). Although this was largely attributable to the fact that positive reference variants had lower scores in the catalytic than in the regulatory domain, this was also true for the random reference variant scores (Mann-Whitney U test, $p = 0.015$). We found that all maps and models performed much better for the catalytic domain (maximum AUBPRC = 0.97) than for the regulatory domain (maximum AUBPRC = 0.78). Although the difference between positive and random variant scores was significant in the catalytic domain (Mann-Whitney U test, $p = 1.65 \times 10^{-4}$), it was only suggestive in the regulatory domain ($p = 0.08$). For both domains, the p.Ala222Val background at 25 μg/mL folinate ("AV25") map was the best performing ($p < 0.001$, see material and methods for test details). Interestingly, among the 78 individuals for which p.Ala222Val status was available, the presence of p.Ala222Val was starkly elevated among homocystinuria cases (OR = 15.7; $p < 2.2 \times 10^{-16}$; Fisher's exact test), explaining why a map in the p.Ala222Val background was the most predictive.

We evaluated the ability of the AV25 map to distinguish positive from random reference variants and compared the results with multiple computational predictors. Examining the regulatory domain separately, the AV25 map showed an AUBPRC of 0.78, which was statistically indistinguishable from that of PROVEAN[46,47] (0.77), PolyPhen-2[48,49] (0.66), SNAP2[50] (0.67), CADD[46,47] (0.81), and Deogen2[48] (0.8). The fraction of positive reference variants that could be recovered at a stringent precision

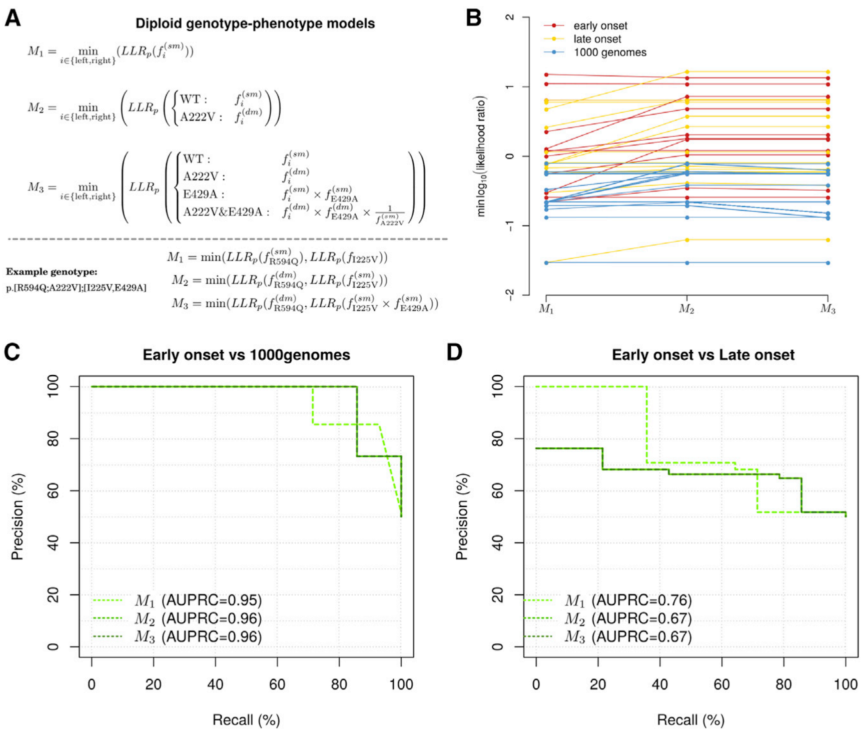

Figure 7. Diploid genotype-to-phenotype prediction

(A) Formal representations of the three tested models with an example variant.

(B) The model predictions for all early-onset, late-onset, and random reference cohorts as they change with increasing model complexity.

(C and D) Precision-recall curve of all models, evaluated on early-onset cases versus random reference individuals (C) and early-onset versus late-onset individuals (D).

threshold (recall at 90% precision, or R90P) was 29% for AV25, which was numerically better than the computational method (R90P was 17%, 0%, 0%, 0%, and 8% for PolyPhen-2, PROVEAN, SNAP2, CADD, and Deogen2, respectively). (see material and methods for details).

For the catalytic domain, the comparison was clearer (see Figure 6C). Here, AV25 achieved an AUBPRC of 0.97, which outperformed every tested computational predictor: AUBPRC values were 0.74, 0.85, 0.76, 0.73, and 0.76 for PolyPhen-2, PROVEAN, SNAP2, CADD, and Deogen2, respectively. Our AV25 map showed an R90P of 75%, which was again higher than was obtained by each of the computational methods: R90P values were 0%, 44%, 11%, 0%, and 33% for PolyPhen-2, PROVEAN, SNAP2, CADD, and Deogen2, respectively. Thus, although the AV25 map performed only on par with computational methods for the regulatory domain, it clearly outperformed computational methods in identifying pathogenic variation in the catalytic domain.

To provide evidence for clinical variant interpretation within a Bayesian framework,[49] we derived a log-likelihood ratio (LLR_p) of pathogenicity for each variant by treating catalytic and regulatory domains separately (see material and methods and Table S2). The respective shapes of the LLR_p functions (Figure S7C) show that, although regulatory domain scores tend to only be useful for predicting pathogenicity, scores in the catalytic domain can provide strong evidence for either pathogenicity or benignity. LLR_p values calculated with our linear models for all possible folinate concentrations up to 200 μg/mL in both backgrounds showed a clear impact for p.Ala222Val on the likelihood of pathogenicity and the dependence of pathogenicity on folinate levels (Figure 6E).

Finally, we compared AV25 map scores with literature-curated measurements of *in vivo* enzyme activities. Although activities measured for the same variants often varied considerably in literature, we nonetheless observed a significant correlation ($r = 0.48$; $p = 9.9 \times 10^{-5}$; Figure S7B). In keeping with our observations in our map, regulatory-domain variants tended to display higher enzyme activity.

Assigning personalized MTHFR functionality scores to diploid genotypes

Of the MTHFR deficiency cases for which we had data (Table S3A), 27 had at least one missense variant. These yielded a positive reference cohort of 15 individuals with early-onset and 12 with late-onset MTHFR deficiency. As a random reference cohort (Table S3B), we used phased diploid genotypes for 77 individuals from the 1000 Genomes Project.[51]

To assign individualized scores to diploid genotypes, we developed a series of three models (Figure 7A; material and methods), differing only in how the presence of multiple *in cis* variants should be handled for calculating an allele score. The first model (M_1) was the most naive, in that it scored only the rare variant in each allele (we saw no instances of multiple *in cis* rare missense variants) and ignored the presence of common variants (p.Ala222Val and p.Glu429Ala). The second model (M_2) exploited our map of genetic interactions with p.Ala222Val for each variant. Finally, the third model (M_3) considered p.Glu429Ala and used the genetic-interaction model to combine the rare variant with p.Ala222Val and a simple product rule to account for additional impacts of p.Glu429Ala. Each model then derived an LLR score for each individual by taking the minimum of each individual's two LLRs of their two alleles *in trans*.

We evaluated each model's ability to distinguish early-onset cases from random reference individuals (Figures

7B and 7C). Changes between M_2 and M_3 were subtle and did not result in any differences in rank order between the two models. Accordingly, their PRC curves were identical, and they both significantly outperformed the naive model M_1 ($p = 0.009$; AUBPRCs 0.96 and 0.96 versus AUBPRC 0.95).

Finally, we wished to evaluate the models in terms of the much more challenging task of distinguishing between early-onset and late-onset cases. Models M_1, M_2, and M_3, yielded statistically indistinguishable ($p > 0.17$ for all pairwise comparisons) AUBPRC performances of 0.76, 0.67, and 0.67, respectively. Model M_1 was significantly better than random guessing ($p = 0.005$), whereas models M_2 and M_3, were still strongly suggestive ($p = 0.071$ and 0.071), despite the fact that their LLR functions had not been calibrated for this distinction.

Discussion

Considering factors influencing the penetrance of genetic variation in *MTHFR* led us to assess the function of variants in different environments (folinate levels) and genetic backgrounds (WT and p.Ala222Val). We were thus able to comprehensively enumerate these effects and show that both folinate and p.Ala222Val substantially modulate the effects of many variants.

Using our atlas, we have gleaned new insight into multiple biochemical mechanisms within MTHFR and specifically into the role of a disordered loop in cofactor retention and the mechanism of SAM-mediated inhibition of MTHFR. The latter also led to our discovery of specific variants that suppress SAM-mediated inhibition and thus lead to hyperactivity of the enzyme.

A number of general observations could be made from the atlas (Figure 3 and Figure S4). For example, variants in the catalytic domain appeared substantially less fit than those in the regulatory domain. Although visually evident in all maps, this could be an artifact of a yeast assay that does not reflect human physiological conditions. The idea that variants in the catalytic region do tend to have stronger impacts in humans is supported by (1) the observation that catalytic-region variants from the gnomAD-derived random reference cohort tended to have lower allele frequencies and to be less often homozygous than those in the regulatory region and (2) by literature-curated enzymatic activity measurements for homocystinemia cases that showed higher activity for regulatory than for catalytic-domain variants.

Most variants in the hydrophobic core, in the active site, and at other molecular interaction interfaces were observed to be functionally abnormal (Figure 1D). Interestingly, residues located at the dimerization interface were not significantly more sensitive to mutation than the remaining surface of the regulatory domain, matching observations that dimerization is not strictly necessary for MTHFR function.[36]

Our atlas offered insight on two novel aspects of MTHFR's biochemistry. A disordered loop stretching across positions 159–174 was implicated as a potential "tether" for an aromatic residue at position 165. This residue is, in the absence of folate, modeled as forming a ⊓-stacking interaction with FAD's flavin group and thus aiding in FAD cofactor retention. Intriguingly, in *E. coli* and *S. cerevisiae,* this loop is shorter (PDB: 6PEY and PDB: 6FNU) and does not contain aromatic residues. The fact that both *E. coli* and *S. cerevisiae*, unlike mammals, are capable of folate biosynthesis and thus do not depend on dietary folate suggests that the origin of this feature is natural selection for an ability to maintain enzyme function at low substrate concentrations.

When examining general trends related to folinate responsiveness, we found hypomorphs more likely to be folinate responsive than both neutral variants and null-like variants (Figure 2D). This mirrors similar observations that were made with respect to vitamin-B_6-responsive variants in the protein cystathionine beta-synthase (CBS) [12] and is consistent with the idea that hypomorphs are more likely to benefit from small-molecule chaperoning.

Another biochemical insight was the (experimentally confirmed) loss of SAM suppression in the Glu463Asn variant, as predicted by hypercomplementation in the atlas. Many variants in the regulatory domain showed hypercomplementation, possibly due to disrupted SAM suppression. Reduced SAM suppression could result from multiple mechanisms, e.g., disruption of the SAM/SAH binding interface, the ability to distinguish between SAM and SAH, the conformational change required to propagate the sensing of SAM, or regulatory-domain stability. However, our atlas scores cannot distinguish among these mechanisms.

This raises the question of what effects (if any) hypercomplementing MTHFR variants may have on human health. Although p.Glu463Asn has not yet been observed in human subjects, two other variants that appear as hypercomplementers in our assay have been seen in late-onset cases of MTHFR deficiency: p.Arg325Cys and p.Val575Gly. The former affects an arginine in close structural proximity to the negatively charged polyglutamylation tail of bound folates. This could conceivably alter enzyme kinetics. However, it is also possible that folate polyglutamylation functions slightly differently in yeast and humans. The latter variant, p.Val575Gly, replaces a valine engaged in multiple hydrophobic interactions in the core of the regulatory domain with a glycine. This might affect the regulatory domain fold, e.g., its stability, to render MTHFR constitutively active.

The atlas revealed interesting patterns of genetic interactions with the common p.Ala222Val variant. The serine-rich region, the inter-domain linker, and the N-terminal halves of both the catalytic and regulatory domains were found to be enriched for genetic interactions. Structurally, these regions are all in direct contact with each other (clustered around the linker) and are likely to play a role in

allosteric conformational change induced by SAM. Additional hotspots of genetic interaction were observed. For example, positions 324–329 form a positive-interaction hotspot atop a TIM barrel alpha helix and near folate's polyglutamyl tail. Most substitutions in this hotspot appeared hypomorphic; many hydrophobic substitutions were strongly folinate remediable, and polar substitutions showed positive genetic interaction with p.Ala222Val, suggesting that these residues control substrate affinity (decreasing affinity would be remediable by higher substrate concentration, whereas heightened folate occupancy would tend to retain FAD and rescue p.Ala222Val). A negative-interaction hotspot can be found at residues 200–207, which form a small loop covering the adenosine group of FAD, and mutations here may decrease FAD retention and thus exacerbate the effects of p.Ala222Val. Although further mechanistic modeling will be required, our map of both positive and negative genetic interactions with p.Ala222Val, exhibiting both folinate-dependent and -independent components, may help distinguish among possible models of the complex interplay between p.Ala222Val and catalytic and regulatory functions.

For the common p.Glu429Ala variant, our map indicated neutrality but also negative genetic interaction with p.Ala222Val. Structurally, Glu429 is located on the surface of the regulatory domain, where its hydrophilic residue is exposed to the surrounding solvent to stabilize MTHFR's regulatory domain. If p.Glu429Ala slightly alters the stability of the regulatory domain, this could explain its negative interaction with p.Ala222Val. However, the *in cis* co-occurrence of these polymorphisms is quite rare (found in none of the homocystinemia cases we considered and in only one individual in the 1000 Genomes control dataset).

Although only early diagnosis and timely therapy[14,16] can prevent long-term complications in individuals with homocystinurias, only a few newborn screening programs worldwide target MTHFR deficiency. This is both because the screening for decreased methionine levels in dried blood spots has low sensitivity[52] and because tHcy screening as a primary marker is expensive. It is therefore worth considering whether future newborn screening programs based on genome sequencing (with subsequent tHcy testing if indicated) could improve the early detection and outcomes for MTHFR deficiency.

In predicting variant pathogenicity, our map scores greatly outperformed a number of computational predictors, especially in the catalytic domain. In the regulatory domain, a few conservation-based predictors, such as PROVEAN and CADD, were able to achieve higher AUBPRCs, but they were not able to achieve a higher recall at 90% precision than the maps. We quantitatively summarized evidence for pathogenicity both for given alleles and for diploid genotypes while accounting for genetic background. We found that the experimental map in the p.Ala222Val background at 25 μg/mL folinic acid performed significantly better than all other maps. This is consistent with the observation that, among the homocystinemia cases' genotypes collected as part of our reference sets, the allele frequency of p.Ala222Val was noticeably elevated (MAF = 41%) relative to the general population (OR = 1.57; $p < 7.5 \times 10^{-3}$; Fisher's exact test). Finally, we found that when considering the full diploid genetic context, models accounting for genetic interactions involving p.Ala222Val significantly improved our performance in classifying subjects by the presence of severe MTHFR deficiency.

The experimental framework we used to generate our MTHFR atlas had some limitations. Altough the yeast-based experimental system performs very well in our benchmark tests with respect to predicting variant pathogenicity, some variant map scores were discordant with their presence in the positive or random (presumed neutral) reference sets, and it is possible that a human-cell-based complementation assay with the potential to capture more organism-specific mechanisms might have made a difference. However, it is difficult to say which (if any) of these cases are failings of the experimental system as opposed to previous misclassifications of reference variants. Examples of discordant variants included the annotated-pathogenic variant p.Ala175Thr (observed as c.523G>A in humans), which exhibited 36% functionality (in the 25 μg/mL folinate p.Ala222Val map that performed best in predicting pathogenicity). Although this indicated reduced function, this score was not low enough for us to predict pathogenicity by using our LLR_p approach. Ala175 is located at the top of an alpha helix within the TIM barrel of the catalytic domain, in close proximity to the FAD's diphosphate. Our atlas provided suggestive evidence (posterior probability = 77%) for a negative genetic interaction with p.Ala222Val. Of the two cases reported to have carried this mutation, the p.Ala222Val status is only known for one of them (heterozygous), but unfortunately without phasing information. Thus, this variant may be pathogenic in an p.Ala222Val-dependent manner, but our assay failed to predict it as such. In another example, the random reference set variant p.His263Pro (observed as c.788A>C in humans) appeared hypomorphic (21% functionality, again in the 25 μg/mL folinate p.Ala222Val map), despite a relatively high MAF of 3×10^{-4} and 36 heterozygous individuals in gnomAD. Although there is no clinical evidence for the pathogenicity of His263 variants, the fact that His263 is located within an alpha helix and forms part of the NADPH and folate binding site and that proline substitutions tend to break helices supports the map-derived suggestion that p.His263Pro is functionally abnormal.

There are many limitations in our ability to provide interpretations for map scores on an absolute scale. We expect that functionality scores are monotonically related to yeast cell fitness; however, the precise relationship remains unknown. Although our scores have been scaled such that scores of 0 correspond to the median of nonsense variants and scores of 1 to the median of synonymous variants, intermediate scores most likely have a non-linear

relationship with cellular fitness, and a (most likely different) non-linear relationship with enzyme activity. A reduced score could result from a variant's impact on total enzyme activity via impacts on specific activity, on protein expression level, or both. Moreover, loss-of-function variants in the regulatory domain affect activity and/or cellular fitness differently than loss of function in the catalytic domain, so that the relationship between the map score, enzyme activity, and cellular fitness might differ between regions. Therefore, our maps might benefit from further calibration of non-linear scaling (e.g., enabled by competitive-growth assays allowing direct and quantitative comparison of the growth impact of variants in different regions), such that intermediate scores from different regions can be made more comparable. However, scaling such that scores in each region correspond to a similar extent of functional loss might not yield the optimal scaling for predicting impacts on human health. This highlights the importance of transforming scores into log-likelihood ratios of pathogenicity (LLR_p), to predict the human impacts of MTHFR variants.

Our underlying variant pool is composed of clones that, in many cases, carry more than one variant. Moreover, variants falling outside of each sequenced tile are not observed. Thus, success of the TileSeq approach requires that each scored variant be present in many independent clones so that the effect of unseen second variants "averages out." This is analogous to the ability of genome-wide association approaches to associate traits with individual variants despite millions of inter-individual differences at other genomic sites. The effects of background variation do tend to average out, as evidenced by the strong separation between synonymous and nonsense variant scores (Figure 1B). Although we cannot be sure that the measures of genetic interaction and folate dependence we derived are robust to unseen variation, our ability to use this data to find and confirm individual mechanistic insights such as those for Trp165 and Glu463 suggest that they are.

In order to model each variant's dependence on folinate and p.Ala222Val status, we used a parsimonious linear model allowing extrapolation to other folinate concentrations. However, the linear model undoubtedly simplifies true behavior and should only be applied within the range of folinate levels tested.

Our complementation assay only assesses MTHFR variants at the level of overall activity. Reduced overall activity could stem from reduced protein stability that lowers total activity while maintaining specific activity or from reduced specific activity without lowered protein abundance. Thus, observation of decreased functionality does not, by itself, provide a mechanistic explanation. Although resolving folate responsiveness and genetic interactions provided additional clues as to these mechanisms, more concrete explanations could be found through the use of additional sub-functional assays, e.g., of protein-protein interaction, protein stability, or responses to regulatory stimuli.

Few variant effect maps have been previously reported,[12,53,54] and here we explored an atlas of dynamic functionality landscapes across combinations of environments and genetic backgrounds. It is worth noting that the most useful map for predicting pathogenicity was measured in 25 μg/mL folinate in the p.Ala222Val background. It would have been impossible *a priori* to predict which of the contexts we examined would be optimal, highlighting the value for future variant effect mapping studies to consider a range of environmental and genetic factors relevant to variant penetrance.

Data and code availability

The TileSeq sequence analysis software can be found on Github at https://github.com/rothlab/tileseq_package (version 1.5). The MAVE scoring pipeline can be found on Github at https://github.com/jweile/tileseqMave (the version at commit number fa8b190 was used). Custom scripts for all downstream analyses can be found on Github at https://github.com/jweile/mthfrModel. The functionality values for all eight experimental variant effect maps have been deposited on MaveDB[55] under accession number urn:mavedb:00000049. Derived maps of basic functionality, folinate-response, folinate-independent genetic interactions and folinate-dependent genetic interactions, as well as lists of variants significantly responding to folinate supplementation or significantly interacting with p.Ala222Val, can be found in Table S2. The literature-curated homocystinemia case genotypes and phenotypes for evaluation and the 1000 Genomes random reference genotypes can be found in Table S3.

Supplemental information

Supplemental information can be found online at https://doi.org/10.1016/j.ajhg.2021.05.009.

Acknowledgments

We took inspiration and advice from J. Rine and appreciate J. Liu and T. Hu for computing infrastructure support. We gratefully acknowledge funding from the One Brave Idea Initiative, the National Human Genome Research Institute of the National Institutes of Health Center of Excellence in Genomic Science Initiative (HG004233 and HG010461), the Canada Excellence Research Chairs, and a Canadian Institutes of Health Research Foundation Grant to F.R.; we also acknowledge a grant by the Czech Health Research Council (NV19-01-00307) and institutional program of the General University Hospital in Prague (RVO-VFN64165) to V.K.. Finally, we are grateful for a grant by the Swiss National Science Foundation (310030_192505) to D.S.F..

Declaration of interests

F.P.R. holds shares in Ranomics and is an investor and advisor for SeqWell (each of which offers services that are potentially useful for variant effect mapping) and has accepted conference travel support from Illumina. S.Y. and R.N. are employees of Invitae, and R.N. is a consultant for Pfizer, Maze Therapeutics, and Genome Medical and a stockholder in Maze Therapeutics and

Genome Medical. The remaining authors declare that they have no competing interests.

Received: January 19, 2021
Accepted: May 18, 2021
Published: July 1, 2021

Web resources

Custom scripts on Github, https://github.com/jweile/mthfrModel
MAVE, https://github.com/jweile/tileseqMave
TileSeq, https://github.com/rothlab/tileseq_package

References

1. van Leeuwen, J., Pons, C., Boone, C., and Andrews, B.J. (2017). Mechanisms of suppression: The wiring of genetic resilience. BioEssays *39*, 1700042.
2. Chen, S., and Parmigiani, G. (2007). Meta-analysis of BRCA1 and BRCA2 penetrance. J. Clin. Oncol. *25*, 1329–1333.
3. Cooper, D.N., Krawczak, M., Polychronakos, C., Tyler-Smith, C., and Kehrer-Sawatzki, H. (2013). Where genotype is not predictive of phenotype: towards an understanding of the molecular basis of reduced penetrance in human inherited disease. Hum. Genet. *132*, 1077–1130.
4. Hartwell, L.H., Szankasi, P., Roberts, C.J., Murray, A.W., and Friend, S.H. (1997). Integrating genetic approaches into the discovery of anticancer drugs. Science *278*, 1064–1068.
5. Aly, A., and Ganesan, S. (2011). BRCA1, PARP, and 53BP1: conditional synthetic lethality and synthetic viability. J. Mol. Cell Biol. *3*, 66–74.
6. Noordermeer, S.M., and van Attikum, H. (2019). PARP Inhibitor Resistance: A Tug-of-War in BRCA-Mutated Cells. Trends Cell Biol. *29*, 820–834.
7. Richards, S., Aziz, N., Bale, S., Bick, D., Das, S., Gastier-Foster, J., Grody, W.W., Hegde, M., Lyon, E., Spector, E., et al.; ACMG Laboratory Quality Assurance Committee (2015). Standards and guidelines for the interpretation of sequence variants: a joint consensus recommendation of the American College of Medical Genetics and Genomics and the Association for Molecular Pathology. Genet. Med. *17*, 405–424.
8. Nykamp, K., Anderson, M., Powers, M., Garcia, J., Herrera, B., Ho, Y.-Y., Kobayashi, Y., Patil, N., Thusberg, J., Westbrook, M., Topper, S.; and Invitae Clinical Genomics Group (2017). Sherloc: a comprehensive refinement of the ACMG-AMP variant classification criteria. Genet. Med. *19*, 1105–1117.
9. Weile, J., and Roth, F.P. (2018). Multiplexed assays of variant effects contribute to a growing genotype-phenotype atlas. Hum. Genet. *137*, 665–678.
10. Weile, J., Sun, S., Cote, A.G., Knapp, J., Verby, M., Mellor, J.C., Wu, Y., Pons, C., Wong, C., van Lieshout, N., et al. (2017). A framework for exhaustively mapping functional missense variants. Mol. Syst. Biol. *13*, 957.
11. Starita, L.M., Ahituv, N., Dunham, M.J., Kitzman, J.O., Roth, F.P., Seelig, G., Shendure, J., and Fowler, D.M. (2017). Variant Interpretation: Functional Assays to the Rescue. Am. J. Hum. Genet. *101*, 315–325.
12. Sun, S., Weile, J., Verby, M., Wu, Y., Wang, Y., Cote, A.G., Fotiadou, I., Kitaygorodsky, J., Vidal, M., Rine, J., et al. (2020). A proactive genotype-to-patient-phenotype map for cystathionine beta-synthase. Genome Med. *12*, 13.
13. Froese, D.S., Huemer, M., Suormala, T., Burda, P., Coelho, D., Guéant, J.-L., Landolt, M.A., Kožich, V., Fowler, B., and Baumgartner, M.R. (2016). Mutation Update and Review of Severe Methylenetetrahydrofolate Reductase Deficiency. Hum. Mutat. *37*, 427–438.
14. Huemer, M., Diodato, D., Schwahn, B., Schiff, M., Bandeira, A., Benoist, J.-F., Burlina, A., Cerone, R., Couce, M.L., Garcia-Cazorla, A., et al. (2017). Guidelines for diagnosis and management of the cobalamin-related remethylation disorders cblC, cblD, cblE, cblF, cblG, cblJ and MTHFR deficiency. J. Inherit. Metab. Dis. *40*, 21–48.
15. Huemer, M., and Baumgartner, M.R. (2019). The clinical presentation of cobalamin-related disorders: From acquired deficiencies to inborn errors of absorption and intracellular pathways. J. Inherit. Metab. Dis. *42*, 686–705.
16. Huemer, M., Diodato, D., Martinelli, D., Olivieri, G., Blom, H., Gleich, F., Kölker, S., Kožich, V., Morris, A.A., Seifert, B., et al.; EHOD consortium (2019). Phenotype, treatment practice and outcome in the cobalamin-dependent remethylation disorders and MTHFR deficiency: Data from the E-HOD registry. J. Inherit. Metab. Dis. *42*, 333–352.
17. Rommer, P.S., Zschocke, J., Fowler, B., Födinger, M., Konstantopoulou, V., Möslinger, D., Stögmann, E., Suess, E., Baumgartner, M., Auff, E., and Sunder-Plassmann, G. (2017). Manifestations of neurological symptoms and thromboembolism in adults with MTHFR-deficiency. J. Neurol. Sci. *383*, 123–127.
18. Watkins, D., Rosenblatt, D.S., and Fowler, B. (2016). Disorders of Cobalamin and Folate Transport and Metabolism. In Inborn Metabolic Diseases: Diagnosis and Treatment, J.-M. Saudubray, M.R. Baumgartner, and J. Walter, eds. (Berlin, Heidelberg: Springer), pp. 385–399.
19. Casas, J.P., Bautista, L.E., Smeeth, L., Sharma, P., and Hingorani, A.D. (2005). Homocysteine and stroke: evidence on a causal link from mendelian randomisation. Lancet *365*, 224–232.
20. Den Heijer, M., Lewington, S., and Clarke, R. (2005). Homocysteine, MTHFR and risk of venous thrombosis: a meta-analysis of published epidemiological studies. J. Thromb. Haemost. *3*, 292–299.
21. Clarke, R., Bennett, D.A., Parish, S., Verhoef, P., Dötsch-Klerk, M., Lathrop, M., Xu, P., Nordestgaard, B.G., Holm, H., Hopewell, J.C., et al.; MTHFR Studies Collaborative Group (2012). Homocysteine and coronary heart disease: meta-analysis of MTHFR case-control studies, avoiding publication bias. PLoS Med. *9*, e1001177.
22. van Meurs, J.B.J., Pare, G., Schwartz, S.M., Hazra, A., Tanaka, T., Vermeulen, S.H., Cotlarciuc, I., Yuan, X., Mälarstig, A., Bandinelli, S., et al. (2013). Common genetic loci influencing plasma homocysteine concentrations and their effect on risk of coronary artery disease. Am. J. Clin. Nutr. *98*, 668–676.
23. Blom, H.J., and Smulders, Y. (2011). Overview of homocysteine and folate metabolism. With special references to cardiovascular disease and neural tube defects. J. Inherit. Metab. Dis. *34*, 75–81.
24. Smulders, Y.M., and Blom, H.J. (2011). The homocysteine controversy. J. Inherit. Metab. Dis. *34*, 93–99.
25. Fezeu, L.K., Ducros, V., Guéant, J.-L., Guilland, J.-C., Andreeva, V.A., Hercberg, S., and Galan, P. (2018). MTHFR 677C → T genotype modulates the effect of a 5-year supplementation with B-vitamins on homocysteine concentration: The SU.FOL.OM3 randomized controlled trial. PLoS ONE *13*, e0193352.

26. Lek, M., Karczewski, K.J., Minikel, E.V., Samocha, K.E., Banks, E., Fennell, T., O'Donnell-Luria, A.H., Ware, J.S., Hill, A.J., Cummings, B.B., et al.; Exome Aggregation Consortium (2016). Analysis of protein-coding genetic variation in 60,706 humans. Nature *536*, 285–291.
27. Karczewski, K.J., Francioli, L.C., Tiao, G., Cummings, B.B., Alföldi, J., Wang, Q., Collins, R.L., Laricchia, K.M., Ganna, A., Birnbaum, D.P., et al. (2019). Variation across 141,456 human exomes and genomes reveals the spectrum of loss-of-function intolerance across human protein-coding genes. bioRxiv, 531210.
28. Guenther, B.D., Sheppard, C.A., Tran, P., Rozen, R., Matthews, R.G., and Ludwig, M.L. (1999). The structure and properties of methylenetetrahydrofolate reductase from Escherichia coli suggest how folate ameliorates human hyperhomocysteinemia. Nat. Struct. Biol. *6*, 359–365.
29. Sibani, S., Leclerc, D., Weisberg, I.S., O'Ferrall, E., Watkins, D., Artigas, C., Rosenblatt, D.S., and Rozen, R. (2003). Characterization of mutations in severe methylenetetrahydrofolate reductase deficiency reveals an FAD-responsive mutation. Hum. Mutat. *21*, 509–520.
30. Shan, X., Wang, L., Hoffmaster, R., and Kruger, W.D. (1999). Functional characterization of human methylenetetrahydrofolate reductase in Saccharomyces cerevisiae. J. Biol. Chem. *274*, 32613–32618.
31. Burda, P., Suormala, T., Heuberger, D., Schäfer, A., Fowler, B., Froese, D.S., and Baumgartner, M.R. (2017). Functional characterization of missense mutations in severe methylenetetrahydrofolate reductase deficiency using a human expression system. J. Inherit. Metab. Dis. *40*, 297–306.
32. Yamada, K., Chen, Z., Rozen, R., and Matthews, R.G. (2001). Effects of common polymorphisms on the properties of recombinant human methylenetetrahydrofolate reductase. Proc. Natl. Acad. Sci. USA *98*, 14853–14858.
33. Liew, S.-C., and Gupta, E.D. (2015). Methylenetetrahydrofolate reductase (MTHFR) C677T polymorphism: epidemiology, metabolism and the associated diseases. Eur. J. Med. Genet. *58*, 1–10.
34. Marini, N.J., Gin, J., Ziegle, J., Keho, K.H., Ginzinger, D., Gilbert, D.A., and Rine, J. (2008). The prevalence of folate-remedial MTHFR enzyme variants in humans. Proc. Natl. Acad. Sci. USA *105*, 8055–8060.
35. Langmead, B., and Salzberg, S.L. (2012). Fast gapped-read alignment with Bowtie 2. Nat. Methods *9*, 357–359.
36. Froese, D.S., Kopec, J., Rembeza, E., Bezerra, G.A., Oberholzer, A.E., Suormala, T., Lutz, S., Chalk, R., Borkowska, O., Baumgartner, M.R., and Yue, W.W. (2018). Structural basis for the regulation of human 5,10-methylenetetrahydrofolate reductase by phosphorylation and S-adenosylmethionine inhibition. Nat. Commun. *9*, 2261.
37. Pejchal, R., Campbell, E., Guenther, B.D., Lennon, B.W., Matthews, R.G., and Ludwig, M.L. (2006). Structural perturbations in the Ala–> Val polymorphism of methylenetetrahydrofolate reductase: how binding of folates may protect against inactivation. Biochemistry *45*, 4808–4818.
38. Benjamini, Y., and Hochberg, Y. (1995). Controlling the False Discovery Rate: A Practical and Powerful Approach to Multiple Testing. J. R. Stat. Soc. Ser. B Stat. Methodol. *57*, 289–300.
39. Suormala, T., Gamse, G., and Fowler, B. (2002). 5,10-Methylenetetrahydrofolate reductase (MTHFR) assay in the forward direction: residual activity in MTHFR deficiency. Clin. Chem. *48*, 835–843.
40. Burda, P., Schäfer, A., Suormala, T., Rummel, T., Bürer, C., Heuberger, D., Frapolli, M., Giunta, C., Sokolová, J., Vlášková, H., et al. (2015). Insights into severe 5,10-methylenetetrahydrofolate reductase deficiency: molecular genetic and enzymatic characterization of 76 patients. Hum. Mutat. *36*, 611–621.
41. Ran, F.A., Hsu, P.D., Wright, J., Agarwala, V., Scott, D.A., and Zhang, F. (2013). Genome engineering using the CRISPR-Cas9 system. Nat. Protoc. *8*, 2281–2308.
42. Shapiro, S.K., and Ehninger, D.J. (1966). Methods for the analysis and preparation of adenosylmethionine and adenosylhomocysteine. Anal. Biochem. *15*, 323–333.
43. Ueland, P.M., and Rozen, R. (2005). MTHFR Polymorphisms and Disease (CRC Press).
44. Frosst, P., Blom, H.J., Milos, R., Goyette, P., Sheppard, C.A., Matthews, R.G., Boers, G.J.H., den Heijer, M., Kluijtmans, L.A., van den Heuvel, L.P., et al. (1995). A candidate genetic risk factor for vascular disease: a common mutation in methylenetetrahydrofolate reductase. Nat. Genet. *10*, 111–113.
45. Goyette, P., and Rozen, R. (2000). The thermolabile variant 677C–>T can further reduce activity when expressed in cis with severe mutations for human methylenetetrahydrofolate reductase. Hum. Mutat. *16*, 132–138.
46. Rentzsch, P., Witten, D., Cooper, G.M., Shendure, J., and Kircher, M. (2019). CADD: predicting the deleteriousness of variants throughout the human genome. Nucleic Acids Res. *47* (D1), D886–D894.
47. Kircher, M., Witten, D.M., Jain, P., O'Roak, B.J., Cooper, G.M., and Shendure, J. (2014). A general framework for estimating the relative pathogenicity of human genetic variants. Nat. Genet. *46*, 310–315.
48. Raimondi, D., Tanyalcin, I., Ferté, J., Gazzo, A., Orlando, G., Lenaerts, T., Rooman, M., and Vranken, W. (2017). DEOGEN2: prediction and interactive visualization of single amino acid variant deleteriousness in human proteins. Nucleic Acids Res. *45* (W1), W201–W206.
49. Tavtigian, S.V., Greenblatt, M.S., Harrison, S.M., Nussbaum, R.L., Prabhu, S.A., Boucher, K.M., Biesecker, L.G.; and ClinGen Sequence Variant Interpretation Working Group (ClinGen SVI) (2018). Modeling the ACMG/AMP variant classification guidelines as a Bayesian classification framework. Genet. Med. *20*, 1054–1060.
50. Hecht, M., Bromberg, Y., and Rost, B. (2015). Better prediction of functional effects for sequence variants. BMC Genomics *16* (*Suppl 8*), S1.
51. Auton, A., Brooks, L.D., Durbin, R.M., Garrison, E.P., Kang, H.M., Korbel, J.O., Marchini, J.L., McCarthy, S., McVean, G.A., Abecasis, G.R.; and 1000 Genomes Project Consortium (2015). A global reference for human genetic variation. Nature *526*, 68–74.
52. Keller, R., Chrastina, P., Pavlíková, M., Gouveia, S., Ribes, A., Kölker, S., Blom, H.J., Baumgartner, M.R., Bártl, J., Dionisi-Vici, C., et al.; individual contributors of the European Network and Registry for Homocystinurias and Methylation Defects (E-HOD) (2019). Newborn screening for homocystinurias: Recent recommendations versus current practice. J. Inherit. Metab. Dis. *42*, 128–139.
53. Stiffler, M.A., Hekstra, D.R., and Ranganathan, R. (2015). Evolvability as a function of purifying selection in TEM-1 β-lactamase. Cell *160*, 882–892.

54. Flynn, J.M., Rossouw, A., Cote-Hammarlof, P., Fragata, I., Mavor, D., Hollins, C., 3rd, Bank, C., and Bolon, D.N. (2020). Comprehensive fitness maps of Hsp90 show widespread environmental dependence. eLife *9*, e53810.

55. Esposito, D., Weile, J., Shendure, J., Starita, L.M., Papenfuss, A.T., Roth, F.P., Fowler, D.M., and Rubin, A.F. (2019). MaveDB: an open-source platform to distribute and interpret data from multiplexed assays of variant effect. Genome Biol. *20*, 223.

The American Journal of Human Genetics, Volume *108*

Supplemental information

Shifting landscapes of human MTHFR missense-variant effects

Jochen Weile, Nishka Kishore, Song Sun, Ranim Maaieh, Marta Verby, Roujia Li, Iosifina Fotiadou, Julia Kitaygorodsky, Yingzhou Wu, Alexander Holenstein, Céline Bürer, Linnea Blomgren, Shan Yang, Robert Nussbaum, Rima Rozen, David Watkins, Marinella Gebbia, Viktor Kozich, Michael Garton, D. Sean Froese, and Frederick P. Roth

Supplemental figures

A

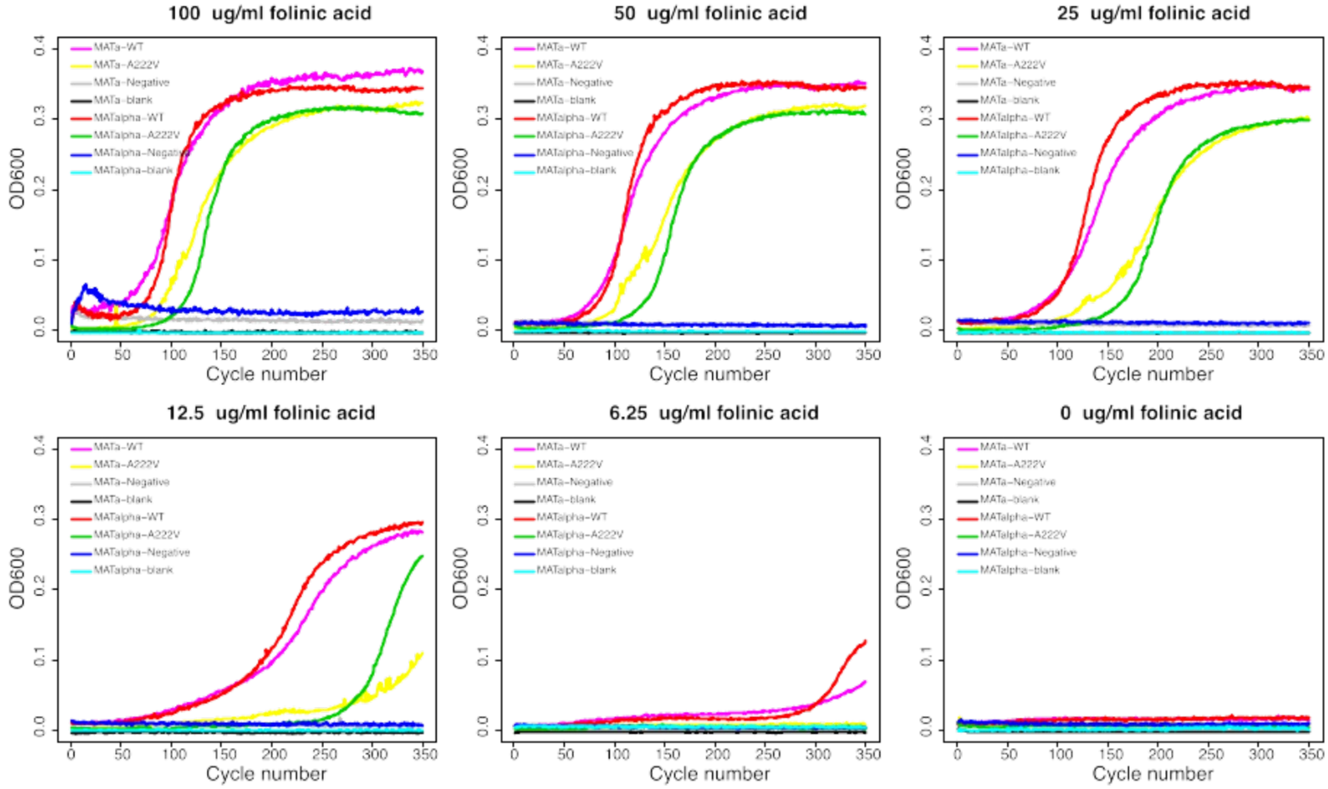

B

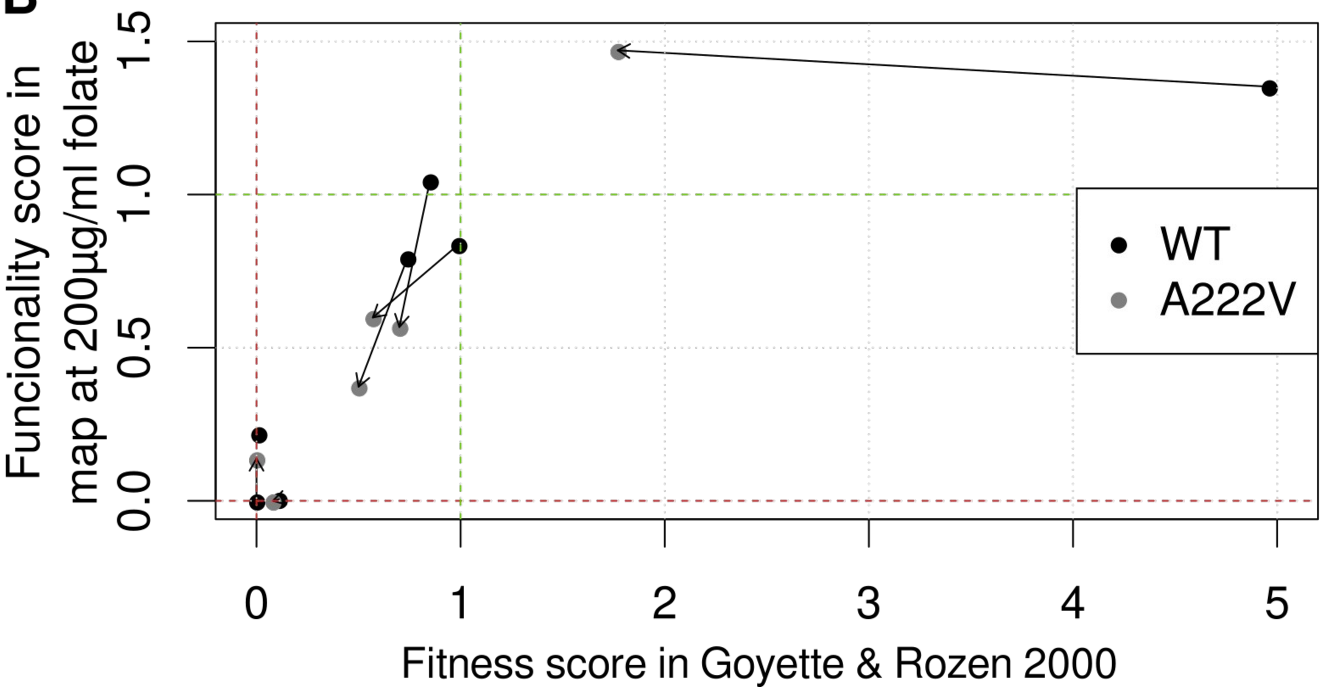

Supplemental Figure S1: A: Assay validation via liquid growth assay. Continuous optical density readings at ƛ=600nm for liquid growth assays of yeast met13-fol3 strains carrying WT human MTHFR,

the p.Ala222Val variant, as well as an empty vector and empty media control at different levels of folinic acid supplementation. p.Ala222Val was confirmed to exhibit a mild functionality defect compared to WT, which increases in severity as the folinic acid concentration decreases. B: Comparison of VE map scores against previous results for relative enzyme activity in WT and p.Ala222Val backgrounds by Goyette and Rozen 2000. Arrows connect corresponding variants in the two backgrounds. Red and green lines correspond to null-like and WT-like functionality, respectively.

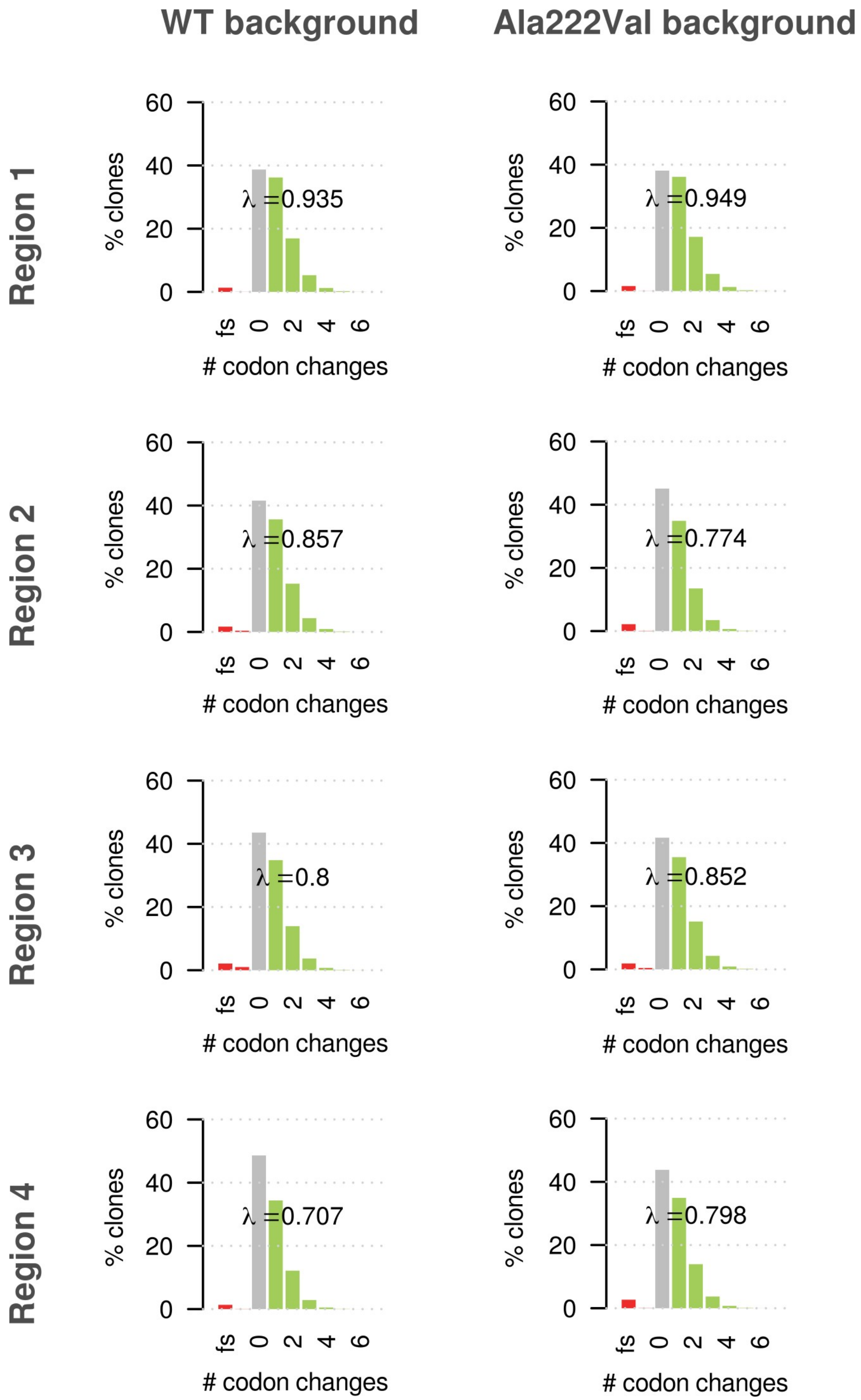

Supplemental Figure S2: Distribution of the number of codon changes across clones in each of the mutagenized libraries. The two red bars on the left indicate the percentage of clones carrying potential frame-shifting and in-frame indels respectively. The Gray bar indicates the percentage of WT clones, while the green bars show clones carrying one or more codon changes. Lambda indicates the

parameter of the best-fitting Poisson distribution over the distribution of codon changes and can be interpreted as the average number of amino acid changes per clone.

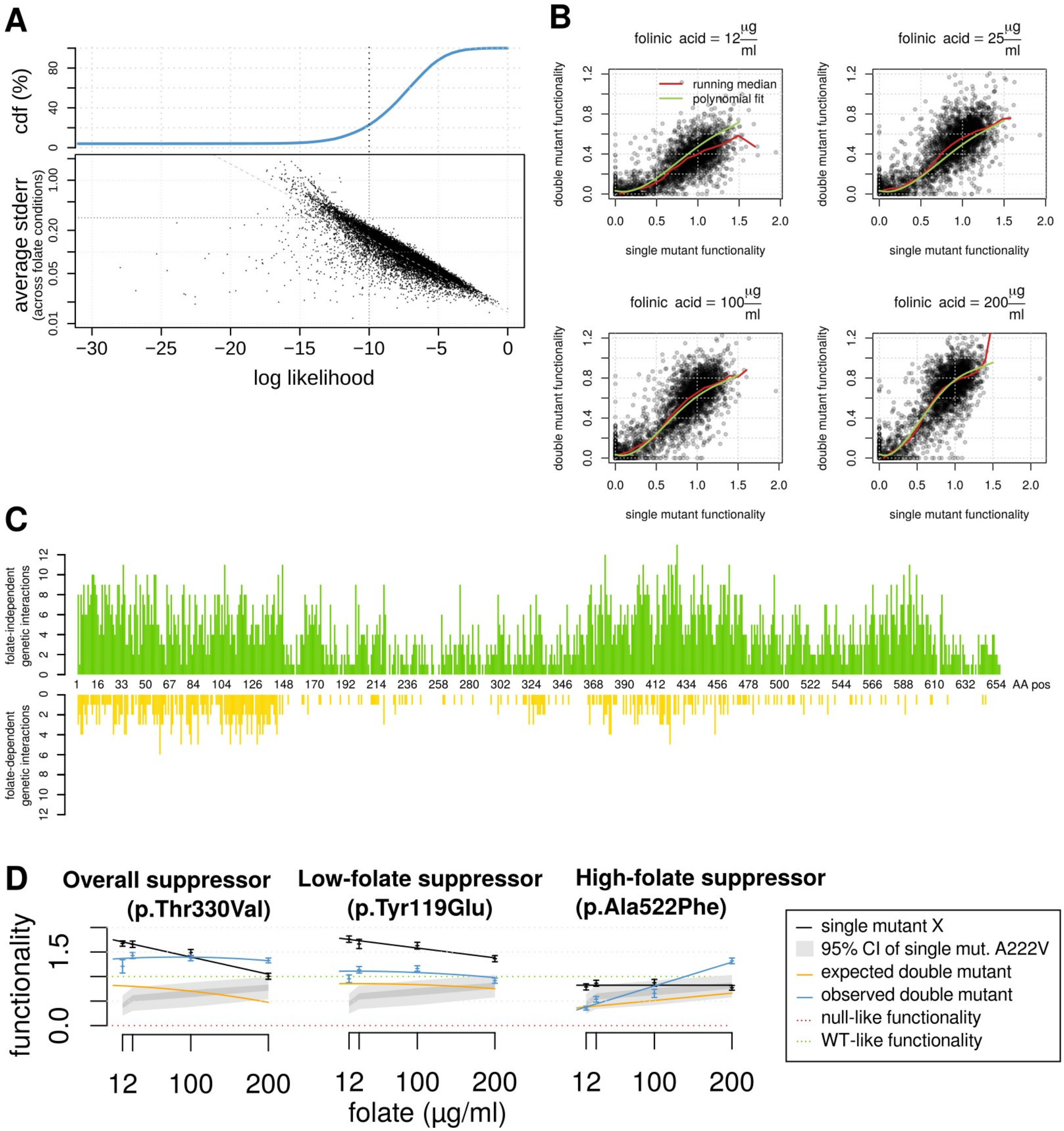

Supplemental Figure S3: A: Cumulative distribution of model log likelihoods and scatterplot comparing log likelihood against the average SEM of modeled data points. B: Running medians and polynomial regression fits for single- vs double-mutant functionality scores at each tested folinate concentration. These form the basis for the “expected double mutant functionality” used to find genetic interactions. C: Number of folate-independent (green) and folate-dependent (yellow) genetic interactions per amino acid position. D: Example variants for three different categories of suppressors of p.Ala222Val. Left: Folate-independent suppressors; Middle: Suppressors at low folate levels; Right:

Suppressors at high folate levels. The black line in each plot shows the single mutant functionality model of the variant in question, with the underlying data points and their SEM indicated by black bars, the gray areas indicate the 95% confidence interval of the p.Ala222Val single mutant functionality based on the distribution of synonymous variants in that background. The orange line indicates the expected double mutant functionality under the regression model. The blue line indicates the best fitting model for the double mutant functionality with the underlying data points and their respective SEM shown as blue bars.

Supplemental Figure S4: Full-sized maps, colors and labels as in Figure 2. See external file MTHFR_map_suppl_fig_S4.png

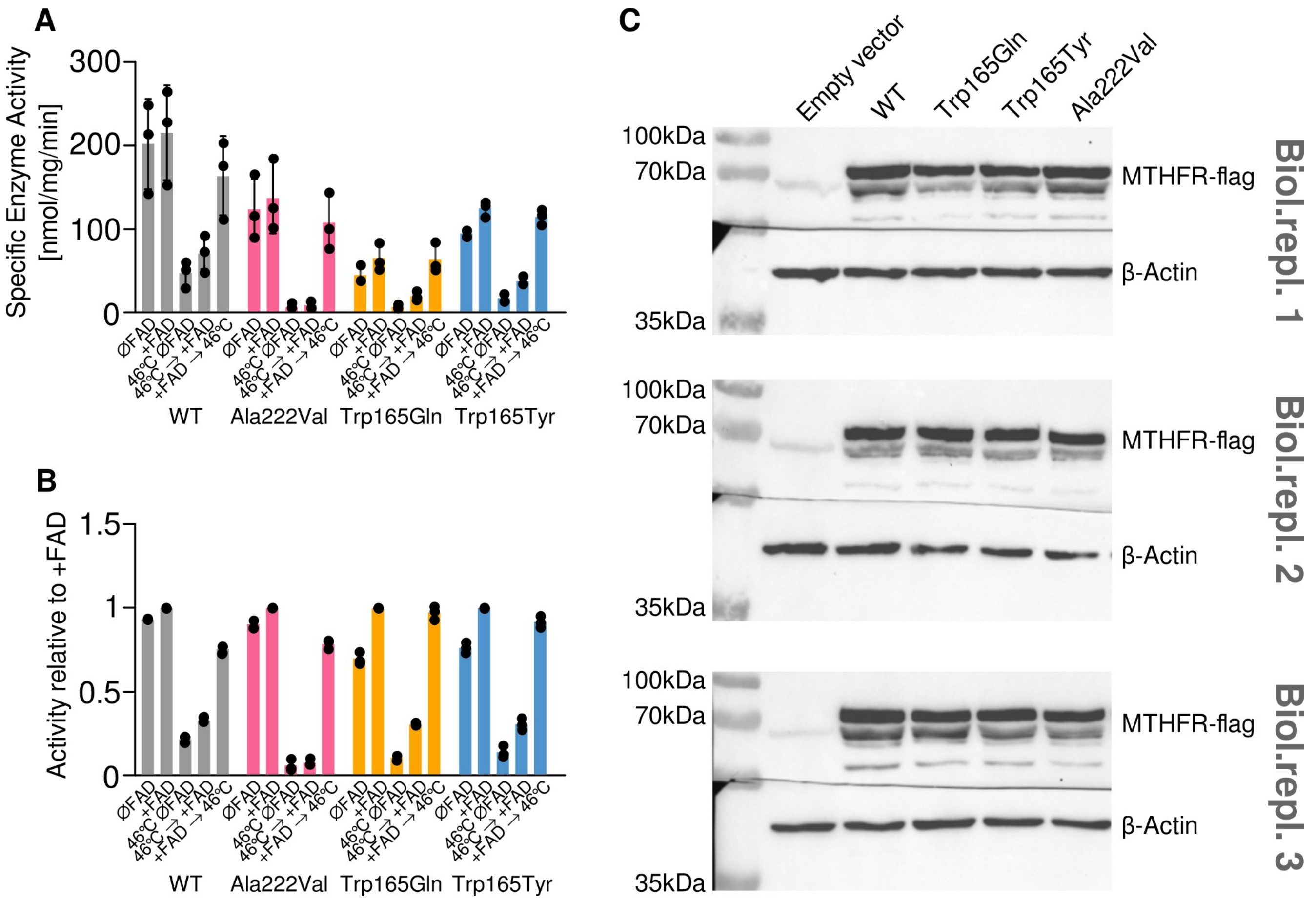

Supplemental Figure S5: Specific enzyme activity as measured by HPLC with cell lysates derived from MTHFR-KO cells following transfection with variant MTHFR vectors. Activity was assayed in the presence and absence of FAD, as well as in three heat treatment conditions: Without FAD, with FAD supplementation before heat treatment and FAD supplementation after heat treatment. (A) Absolute specific activity. Error bars show standard deviation. (B) Activity normalized relative to +FAD condition. Error bars show standard deviation. (C) Western blots confirm the expression levels of the tested variants.

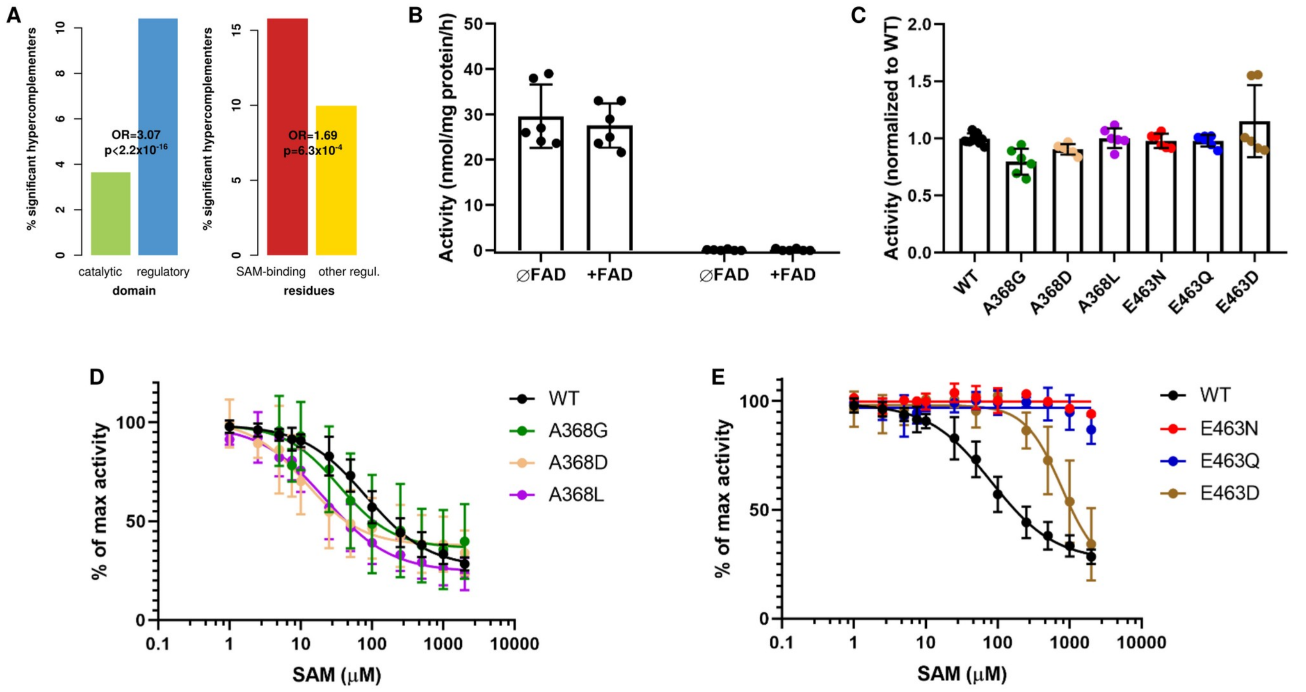

Supplemental Figure S6: A. Fisher's exact tests for enrichment of hypercomplementers in the regulatory domain, as well as enrichment of hypercomplementers in residues near the SAM binding interface. B. Activity of endogenous MTHFR from cell lysates of unmodified HEK293T (ATCC: CRL-3216, WT) cells and genetically engineered MTHFR knock-out HEK293 cells (MTHFR-KO). Assay was performed in the absence (ØFAD) and presence (+FAD) of 75 µM FAD supplemented to the reaction mixture. n = 3 biological replicates, performed in duplicate. C. Maximum activity of MTHFR from cell lysates derived from MTHFR-KO cells following transfection with WT or mutant MTHFR vectors. To account for activity variability following transfection (REF: Burda et al. J Inherit Metab Dis 2016), activity is normalized to WT for each of n = 3 biological replicates (performed in duplicate). D and E. SAM inhibition of MTHFR from cell lysates derived MTHFR-KO cells following transfection with WT or mutant vectors. n = 3 biological replicates (performed in duplicate). Ki's: WT: 82.4 ± 10.4 µM; A368G: 35.0 ± 16.4 µM; A368D: 37.8 ± 4.0 µM; A368L: 24.3 ± 5.3 µM; E463N: n.d.; E463Q: n.d.; E463D: n.d. where n.d. means not determinable. Error bars in B-E show standard deviation.

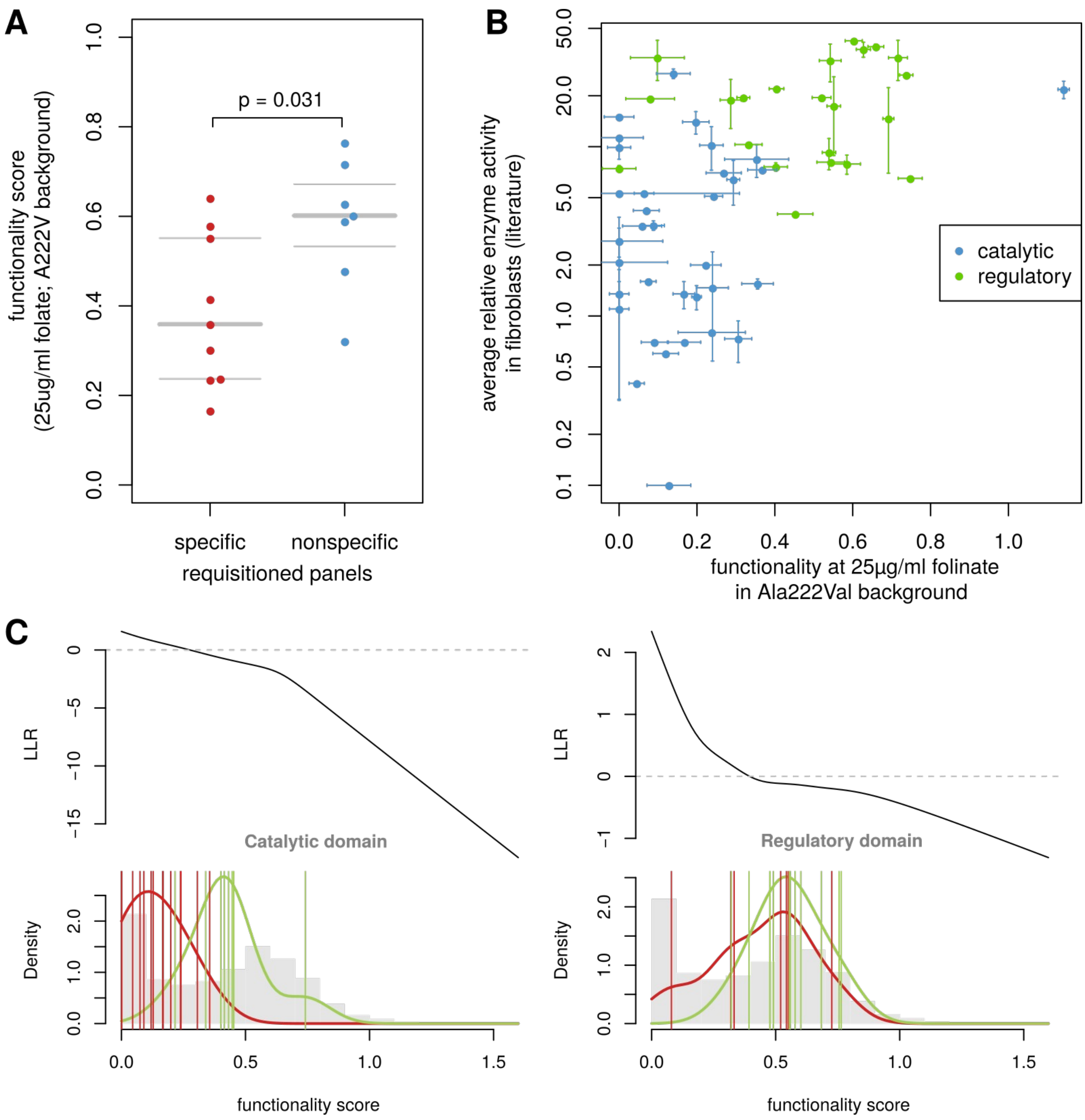

Supplemental Figure 7: A: Functionality scores in the VE map for 25µg/ml folate in the p.Ala222Val background for variants observed by Invitae in disease-specific and non-specific panels. p-value shown for Mann-Whitney U-test. B: Functionality at 25µg/ml folinic acid in the p.Ala222Val background correlates with average enzymatic activity in fibroblasts as reported in the literature (see Methods). Error bars show standard deviation. Blue data points are catalytic domain variants, while green data points are regulatory domain variants. C: Transformation functions from functionality scores to log likelihood ratios of pathogenicity in the catalytic and regulatory domains. The functions express the log ratio between the likelihood of observing a given score in the score distribution of the positive reference (red) set as opposed to that of the negative reference set (green). Gray histogram bars show the distribution of missense variants for comparison.

Supplemental Tables

Background	folinic acid (µg/ml)	Possible AA variants	detected	%	passed filter	%
WT	12.5	13776	13190	95.75%	12534	90.98%
WT	25	13776	13189	95.74%	12494	90.69%
WT	100	13776	13191	95.75%	12530	90.96%
WT	200	13776	13142	95.40%	12551	91.11%
p.Ala222Val	12.5	13776	12970	94.15%	12011	87.19%
p.Ala222Val	25	13776	12970	94.15%	11997	87.09%
p.Ala222Val	100	13776	12964	94.11%	12061	87.55%
p.Ala222Val	200	13776	12916	93.76%	12158	88.25%

Supplemental Table S1: Filter pass rates for all possible AA change, synonymous and nonsense variant across all 8 conditional maps. Detected: Variants for which at least one sequencing read was detected. Passed filter: Variants that passed the quality filtering procedure (see Methods for details) and were accepted into the map.

Supplemental Table S2: Variant effect data and models of functionality, folinate response and genetic interactions. See file MTHFR_map_suppl_tableST2.xlsx

Supplemental Table S3: Curated reference variant sets for validation. See file MTHFR_map_suppl_tableST3.xlsx

Supplemental Table S4: List of TileSeq primers and PopCode mutagenesis oligos. See file MTHFR_map_suppl_tableST4.xlsx

Supplemental Methods

A. Analysis of sequence data to derive raw functionality scores

After consolidating variants by amino acid change outcome, variants with a frequency of pre-selection reads that fell below three standard deviations above the replicate mean of the corresponding non-mutagenized control were considered to have a frequency indistinguishable from that arising due to PCR or base-calling errors and filtered out. Similarly, variants with fewer post-selection read counts than this threshold were considered lost in a culture propagation bottleneck and also filtered out. The dataset was split into sets of variants falling into the original mutagenesis regions, so they could be rescaled separately from each other. Frequencies in the non-mutagenized control frequencies were then subtracted from pre- and post-selection frequencies to adjust for sequencing cycle-specific error biases. The raw functionality scores were then calculated as the log ratio between the corrected pre- and post selection counts. The final scores were then calculated by adjusting the functionality scores to a 0 to 1 scale, where 0 corresponds to the median score of nonsense variants (assumed to be complete loss of function) and 1 corresponds to the median score of synonymous variants (assumed to be of WT-like function). Importantly, this is done for each mutagenesis region separately, to ensure that they fall on the same scale.

Measurement uncertainty was determined by using a method by Baldi and Long[55] to regularize the empirical coefficient of variation across technical replicates using a prior obtained from linear regression against the raw read counts. Error was propagated through each subsequent transformation operation using Taylor approximations. The full code for performing these steps can be found on Github at https://github.com/jweile/tileseqMave (commit number fa8b190).

B. Modeling dependence of variant effects on folinate and p.Ala222Val

Given only four data points per variant (i.e. the measurements taken at the four chosen folinate concentrations), modeling functionality in terms of folinate supplementation required a parsimonious approach. Evaluating both linear and sigmoid models using Akaike's Information Criterion (AIC), we found 96.1% of variants to have a more favorable (lower) AIC under the linear model. Manual inspection of maximum-likelihood sigmoid fits also revealed that these tended to either mimic linear behavior or assume extreme step-like shapes.

Given these results, the response of each variant (i) to folinate supplementation was modeled using a simple linear function that expresses functionality at concentration c in terms of a base functionality (b) and a folinate response parameter (r).

$$f_i^{(sm)}(c) = b_i + r_i c$$

The likelihood of a given model can be determined as the product of densities under normal distributions based on the sample means and standard deviations of the experimental measurements ($\hat{f}_{i,c}$ and $\hat{\sigma}_{i,c}$) at the four given folinate concentrations:

$$\mathcal{L}\left(f_i^{(sm)}(c)\right) = \prod_{c\in\{12.5,25,100,200\}} \varphi_{\mu=\hat{f}_{i,c},\sigma=\hat{\sigma}_{i,c}}\left(f_i^{(sm)}(c)\right)$$

We determined the maximum likelihood model for each model using the Nelder-Mead algorithm implemented in the “optimization” R package[1,2].

To find a threshold for acceptable model quality, we compared the log likelihood against the average experimental standard error across conditions. We found that a model log likelihood of -10 roughly corresponded to a maximal standard error of 0.2 in terms of functionality (where a unit of 1 represents the difference between WT and complete loss of function).

To assess the plausibility of each response model we compared it with the corresponding null-model, which assumes no supplementation response (calculating the likelihood that the true functionality is constant at the mean of all four measurements, given their respective measurement error). This null-model likelihood allows for the determination of a log likelihood ratio (LLR_s) expressing how much more likely the supplementation response model is compared to the null model. To correct for multiple hypothesis testing and limit the false positive rate, we applied a prior probability of 1% and selected those cases in which the LLR_s transformed this prior to a posterior probability of greater than 99%.

To model dependence of each variant on the p.Ala222Val background, a similar model was used. We modeled the expectation for double mutant functionality $f^{(edm)}$ as a third order polynomial spline interpolation between single and double-mutant fitness given each folinate concentration and the functionality of p.Ala222Val itself at the same concentration. Folinate-independent and folinate-dependent genetic interactions were then modeled as additive parameters $\varepsilon^{(b)}$ and $\varepsilon^{(r)}$:

$$f_i^{(dm)}(c) = f^{(edm)}\left(f_i^{(sm)}, f_{\text{A222V}}^{(sm)}, c\right) + \varepsilon_i^{(b)} + \varepsilon_i^{(r)} c$$

Model likelihood was calculated as for the single mutant functionality scores. Plausibility of models was evaluated against two different null models, one without genetic interactions (i.e. $\varepsilon^{(b)} = \varepsilon^{(r)} = 0$), and the other modeling only folinate-independent genetic interactions (i.e. $\varepsilon^{(r)} = 0$). LLR_g values were calculated as above for LLR_s.

C. Molecular dynamics simulation of the FAD binding site

Using above-described structural model (created from PDB:6FCX and PDB:2FMN) we used Amber Modeller 9.24 to add the amino acids for the missing disordered loop across positions 159-174. We created six different models, with residue 165 represented as either the WT tryptophan, or a mutant aromatic tyrosine or a mutant polar glutamine; each in the presence and absence of a docked LY309887 (a folate analogue) at the active site. The Antechamber

package[3] was used to generate topology and coordinate files containing FAD and LY309887. Using Amber18[4], we neutralized the models using Cl^- and Na^+ ions and solvated using the TIP3P water model (buffer distance 12Å). Using periodic boundary conditions, we then sequentially performed steepest-descent and conjugate-gradient-energy minimization, followed by equilibration molecular dynamics simulations, gradually removing constraints and heating from 0 to 300K. Unconstrained molecular dynamics simulations were then performed on the equilibrated systems using Amber18. We calculated 10 replicate trajectories for each model, across a timeframe of 200 ns with a resolution of 0.2 ns.

We then calculated the distance between the alpha carbon atom of amino acid 165 (i.e. the WT Trp, or mutant Tyr or Gln) and the C1 carbon of the FAD flavin (using Euclidean distance at each simulation time point). We next calculated the distance of between FAD's central N5 atom from the center of the top of the catalytic domain's TIM-barrel (defined as as the arithmetic centroid between the alpha carbons of residues 321, 256, 227, 196, 156, 129, 93, and 64), also at every time point. These distance trajectories were then used to calculate the time spent at given distances.

To generate the Markov Model of interaction states between Trp165 and the FAD flavin, we calculated the relative transposition vector and relative rotation quaternion between the two molecules' respective aromatic rings in each time point and then used Mclust[5] to perform Gaussian mixture model clustering, identifying 8 distinct clusters. We calculated the centroid of each cluster and visualized the timepoints with the greatest similarity to each centroid using OpenPyMol[6]. Finally, we examined the pattern in which the simulation trajectories traversed through cluster members and calculated lingering times and transition probabilities. These were then used to construct the state transition model.

D. Compilation of reference variant sets

To evaluate the ability of map scores to predict variant pathogenicity (and ultimately individual phenotypes), we needed to establish positive and negative variant reference sets. Hyperhomocysteinemia case genotypes and phenotypes were assembled largely from previous publications[7,8]. For all individuals for which data had not been published previously: clinical, biochemical, and molecular genetic data were obtained during routine care; individuals gave their informed consent for DNA analysis; and phenotypes were collected within a research project after obtaining informed consent, which included also a consent for the publication of clinical, enzymatic, and molecular genetic data. For individuals followed in the Metabolic Center in the Department of Pediatrics and Adolescent Medicine, the General University Hospital in Prague, there was approval of the Ethics Committee (1194/13 S-IV). All data collection conformed to the principles of the Helsinki Declaration.

We integrated these datasets to match the most recent MTHFR reference sequence and converted the associated phenotype data to use uniform time units for age of onset. The dataset (Supplemental Table S3A) comprises 206 samples, 197 and 128 of which are labeled approximate and detailed age of onset, respectively. All but two samples provide genotype

information, with 89 of them carrying missense variants in both alleles. However only 78 samples provide information on p.Ala222Val status.

We extracted the list of missense variants in the above dataset and tallied how often they each occured in early- and late-onset cases respectively. Following existing convention, the classification cutoff for early onset was defined as diagnosis no later than 12 months of age[7,8]. Variants that were seen more often in early onset than late onset cases were included in the "early onset positive reference set" (comprising 30 variants); while variants that were more often observed in late onset cases than early onset cases were included in the "late onset reference set" (comprising 40 variants). Ties were excluded.

Next, to obtain a random reference cohort, we accessed gnomAD[9,10] (a collection of genotypes meant to be comprised primarily of unaffected individuals), filtering for missense variants in MTHFR that fulfilled either one of the two following criteria to enrich for variants likely to be benign: (i) The global minor allele frequency is above 1 in 10,000; or (ii) at least one homozygous case has been observed. Within this set of variants set we found that two gnomAD entries (c.1408G>C = p.Glu470Gln and c.1409A>T = p.Glu470Val) were actually a mis-annotated multi-nucleotide variant (c.1408_1409delinsCT = p.Glu470Leu). We therefore replaced these variants with the correct MNV in our resulting random reference set. We also removed p.Ala222Val, as it was already used here as a common genetic background for our maps.

E. Sequencing panel enrichment analysis

We examined MTHFR variants previously observed in clinical sequencing by Invitae. Although phenotypes are not available for sequenced individuals, it is known which gene panel was requested by the physician for sequencing. Variants were stripped of all protected health information (i.e. de-identified) under an approved protocol from the Western Institutional Review Board (IRB #20161796). Panel-associations were collated for each unique variant and were classified into two categories: Relevant disease-specific panels (homocystinuria, fatty-acid oxidation defects and neurometabolic disease) and Nonspecific panels (such as carrier screening). We then grouped variants according to whether they were more often seen in specific or nonspecific panels and compared the distributions of corresponding functionality values from our atlas (using the 25µg/ml folinic acid and p.Ala222Val background map) via Mann-Whitney-U test.

F. Determining a log likelihood ratio of pathogenicity for each variant

The evaluation against reference sets showed that functionality scores in the catalytic and regulatory domains were not on a comparable scale in terms of predicting disease. After all, the numerical values in our atlas relate to to the fitness of yeast cells as a result of the degree of functionality of the human variant in the host pathway. Therefore, we implemented a transformation function to represent variant effects in terms of the strength of evidence towards and against pathogenicity, that is, a log likelihood ratio of pathogenicity.

To this end we used the distributions of functionality scores for the positive and random reference sets in both domains in the best-performing map (p.Ala222Val background at 25µg/ml folinate) and the best-performing metaparameter for the linear model (WT background at 120µg/ml folinate) to construct likelihood ratios. For any given score, we calculate how much more (or less) likely it is to observe it under the distribution of the positive reference set than under the distribution of the random reference set. We determined the probability density functions for functionality scores in the positive (early onset) reference variants and negative (gnomAD) reference variants in each domain using kernel density estimation via the R package kdensity[11]. The log likelihood ratio (LLR_p) transformation for a given functionality f was then defined as:

$$LLR_p(f) = \log\left(\frac{\pi_X(f)}{\pi_Y(f)}\right)$$

, where π_X is the probability density of the positive reference set distribution and π_Y is the probability density of the random reference set distribution.

G. Modeling individualized diploid genotypes

The first model (M_1) interprets variants as if they were in the WT background; the second model, (M_2) accounts for p.Ala222Val. The third model (M_3) accounts for an additional common variant, p.Glu429Ala (E429A). All models transform functionality scores to pathogenicity LLRs and then use the minimum LLR across variants occurring in trans (in keeping with the recessive mode of inheritance)

$$M_1 = \min_{i \in \{\text{left},\text{right}\}} (LLR_p(f_i^{(sm)}))$$

$$M_2 = \min_{i \in \{\text{left},\text{right}\}} \left(LLR_p \left(\begin{cases} \text{WT}: & f_i^{(sm)} \\ \text{A222V}: & f_i^{(dm)} \end{cases} \right) \right)$$

$$M_3 = \min_{i \in \{\text{left},\text{right}\}} \left(LLR_p \left(\begin{cases} \text{WT}: & f_i^{(sm)} \\ \text{A222V}: & f_i^{(dm)} \\ \text{E429A}: & f_i^{(sm)} \times f_{\text{E429A}}^{(sm)} \\ \text{A222V\&E429A}: & f_i^{(dm)} \times f_{\text{E429A}}^{(dm)} \times \frac{1}{f_{\text{A222V}}^{(sm)}} \end{cases} \right) \right)$$

H. Prior-balancing and significance testing for precision-recall analysis

The precision observed at a given score threshold is defined as the number of true positive calls divided by the total number of positive calls. As the threshold increases in stringency, the

total number of positives decreases, leading to reduced numerical stability and increased uncertainty for precision estimates. This can be expressed as a Bernoulli process, governed by a binomial distribution for *i* true positives out of *n* total positives:

$$\mathbb{P}(i|\rho) = \binom{n}{i}\rho^i(1-\rho)^{(n-i)}$$

Thus, if we model a true precision ρ that is lower than x, then the likelihood of the observation is:

$$\mathbb{P}(i|\rho < x) = \int_0^x \binom{n}{i}\rho^i(1-\rho)^{(n-i)}d\rho$$

However, since we are interested in the CDF of the posterior P(ρ<x|i), we need to use Bayes' theorem.

$$\mathbb{P}(\rho < x|i) = \frac{\mathbb{P}(i|\rho < x)\mathbb{P}(\rho < x)}{\mathbb{P}(i)}$$

Then, using a uniform prior:

$$\mathbb{P}(\rho < x|i) = \frac{\int_0^x \binom{n}{i}\rho^i(1-\rho)^{(n-i)}d\rho \cdot x}{\int_0^1 \binom{n}{i}\rho^i(1-\rho)^{(n-i)}xd\rho} = \frac{\int_0^x \binom{n}{i}\rho^i(1-\rho)^{(n-i)}d\rho}{\int_0^1 \binom{n}{i}\rho^i(1-\rho)^{(n-i)}d\rho}$$

To estimate p-values for significant differences in AUPRC between two predictors, we can calculate the CDF for the reference predictor as above, then calculate the AUC at each quantile of the above distribution and look up the AUC of the second predictor in the CDF of the first. This yields the probability of observing an AUC at least as extreme. An R-implementation of this can be found at https://github.com/jweile/yogiroc.

To enforce the tendency for increasingly stringent thresholds to yield increased precision (at the cost of recall), we apply a 'monotonization' function to the PRC curve, such that precision levels (and confidence interval traces) observed at a given stringency can only rise or stay constant as stringency is further increased.

Imbalances in the sample sizes of the positive and negative reference sets lead to different underlying prior probabilities (of pathogenicity in this case). Varying priors make it difficult to compare precision recall curves with one another, as precision of a prediction is a function of both the strength of evidence and the prior probability that the prediction will be correct. We therefore generated balanced precision vs. recall curves as in Wu et al (under review), via a procedure derived from Bayes' Rule. Briefly, if *ρ* is the precision at a given score threshold, and *P* the prior probability, then the balanced precision is:

$$\rho_{\text{balanced}} = \frac{\rho(1-P)}{\rho(1-P)+(1-\rho)P}$$

Supplemental references

1. Nelder, J.A., and Mead, R. (1965). A Simplex Method for Function Minimization. Comput. J. *7*, 308–313.

2. Husmann, K., Lange, A., and Spiegel, E. (2017). The R Package optimization : Flexible Global Optimization with Simulated-Annealing. p.

3. Wang, J., Wang, W., Kollman, P.A., and Case, D.A. (2006). Automatic atom type and bond type perception in molecular mechanical calculations. J. Mol. Graph. Model. *25*, 247–260.

4. Case, D.A., Cheatham, T.E., Darden, T., Gohlke, H., Luo, R., Merz, K.M., Onufriev, A., Simmerling, C., Wang, B., and Woods, R.J. (2005). The Amber biomolecular simulation programs. J. Comput. Chem. *26*, 1668–1688.

5. Scrucca, L., Fop, M., Murphy, T.B., and Raftery, A.E. (2016). mclust 5: Clustering, Classification and Density Estimation Using Gaussian Finite Mixture Models. R J. *8*, 289–317.

6. Schrödinger (2016). The PyMOL Molecular Graphics System.

7. Froese, D.S., Huemer, M., Suormala, T., Burda, P., Coelho, D., Guéant, J.-L., Landolt, M.A., Kožich, V., Fowler, B., and Baumgartner, M.R. (2016). Mutation Update and Review of Severe Methylenetetrahydrofolate Reductase Deficiency. Hum. Mutat. *37*, 427–438.

8. Ueland, P.M., and Rozen, R. (2005). MTHFR Polymorphisms and Disease (CRC Press).

9. Lek, M., Karczewski, K.J., Minikel, E.V., Samocha, K.E., Banks, E., Fennell, T., O'Donnell-Luria, A.H., Ware, J.S., Hill, A.J., Cummings, B.B., et al. (2016). Analysis of protein-coding genetic variation in 60,706 humans. Nature *536*, 285–291.

10. Karczewski, K.J., Francioli, L.C., Tiao, G., Cummings, B.B., Alföldi, J., Wang, Q., Collins, R.L., Laricchia, K.M., Ganna, A., Birnbaum, D.P., et al. (2019). Variation across 141,456 human exomes and genomes reveals the spectrum of loss-of-function intolerance across human protein-coding genes. BioRxiv 531210.

11. Moss, J., and Tveten, M. (2019). kdensity: Kernel Density Estimation with Parametric Starts and Asymmetric Kernels.
